# Aptamer–ODN Chimeras: Enabling Cell-Specific ODN Targeting Therapy

**DOI:** 10.3390/cells14100697

**Published:** 2025-05-12

**Authors:** Bei Xia, Qubo Zhu

**Affiliations:** Xiangya School of Pharmaceutical Sciences, Central South University, Changsha 410013, China; 227211047@csu.edu.cn

**Keywords:** aptamer, oligonucleotides, siRNA, ASO, miRNA

## Abstract

Oligonucleotides (ODNs) such as siRNA, saRNA, and miRNA regulate gene expression through a variety of molecular mechanisms and show unique potential in the treatment of genetic diseases and rare diseases, but their clinical application is still limited by the efficiency of the delivery system, especially the problem of the insufficient targeting of extrahepatic tissues. As homologous nucleic acid molecules, aptamers have become a key tool to improve the targeted delivery of ODNs. Aptamer–ODN chimeras can not only bind to multiple proteins on the cell surface with high specificity and selectivity, but they can also internalize into cells. Furthermore, they outperform traditional delivery systems in terms of cost-effectiveness and chemical modification flexibility. This review systematically summarizes the origin and progress of aptamer–ODN chimera therapy, discusses some innovative design strategies, and proposes views on the future direction of aptamer-ODN chimeras.

## 1. Introduction

Oligonucleotides (ODNs) such as small interfering RNA (siRNA), small activating RNA (saRNA), and microRNA (miRNA) are nucleic acid polymers that exhibit significant therapeutic potential for the treatment and management of various diseases. Their mechanisms of action primarily involve the precise regulation of gene expression through multiple molecular pathways, including but not limited to RNA interference (RNAi), RNase H-mediated cleavage, alternative splicing regulation, programmed gene editing, and gene activation [1]. They exhibit target specificity through two distinct interaction modalities: the majority act through canonical Watson–Crick base pairing with complementary nucleic acid targets to achieve sequence-specific recognition, while a minority, exemplified by aptamers and small guiding RNAs (sgRNAs), interact with proteins by forming three-dimensional secondary structures [1]. Compared to traditional drugs, ODN-based therapeutics have the potential to target previously undruggable pathogenic genes and patient-specific sequences associated with rare diseases [2]. As of March 2025, a total of 20 ODN-based therapeutic products have received regulatory approval from the US Food and Drug Administration (FDA) and the European Medicines Agency (EMA) for the treatment of a diverse range of diseases [3]. Despite the considerable progress in the clinical application of ODN-based therapeutics, the lack of effective delivery methods, particularly for targeted extrahepatic tissue distribution and tissue-specific cell surface receptor engagement, remains a critical translational barrier in the field. Numerous combinatorial strategies have been developed to enhance ODN delivery to target cells, including but not limited to lipid-based nanoparticles, peptide conjugates, and inorganic nanocarriers [4,5,6]. Furthermore, the conjugation of therapeutic ODN with nucleic acid aptamers has been extensively investigated as a promising approach to improve cell-specific targeting and intracellular delivery efficiency [7].

**Figure 1:**
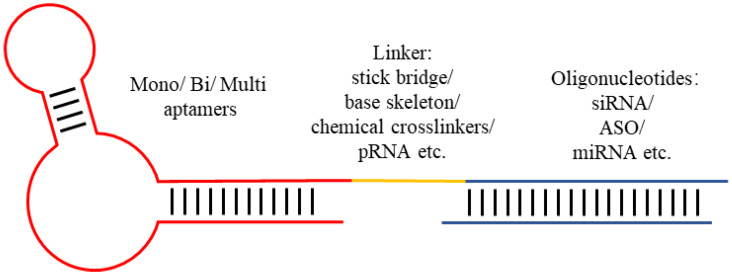
Schematic illustration of the design of aptamer–ODN chimeras.

Since aptamers are also composed of nucleotides, they exhibit the same chemical and biological properties as ODNs such as a base pairing ability, chemical modification compatibility, etc. [8]. Compared to other ODNs, aptamers demonstrate a dual functional advantage: they can not only specifically bind to their targets but also be internalized into cells after binding, making them a powerful vehicle for the targeted delivery of ODNs [7]. Through molecular engineering techniques, aptamers have been extensively explored for programmable and facile coupling with diverse therapeutic ODNs, enabling their targeted delivery (Figure 1) [9]. Aptamer–ODN chimeras show superior advantages over other ODN delivery methods in terms of production cost, flexibility in chemical modification, and safety [10].

This review systematically summarizes the origin and progress of aptamer–ODN chimera therapy, discusses innovative design strategies, and proposes views on the future direction of aptamer-ODN chimeras.

## 2. Aptamers

### 2.1. Introduction and Application

Aptamers are synthetic, short (approximately 20–100 nucleotides in length), single-stranded DNA or RNA molecules that fold into defined three-dimensional structures, enabling them to bind target molecules with high specificity and affinity [11]. They are selected through an iterative in vitro process known as the Systematic Evolution of Ligands by Exponential Enrichment (SELEX). The classical SELEX technique involves the following key steps: A highly diverse random ODN library containing approximately 10^14^ to 10^16^ unique nucleic acid molecules is constructed. The library is incubated with the target molecule, allowing specific nucleic acid sequences to bind to the target. Through methods such as elution and affinity purification, the ODNs that bind to the target are separated from the rest of the library and then amplified using PCR (for DNA libraries) or RT-PCR (for RNA libraries) to obtain enriched sequences. The amplified ODNs are re-incubated with the target molecule, and the binding, separation, and amplification steps are repeated. Typically, 6 to 20 rounds of selection are performed to progressively enrich the sequences with high affinity and specificity. The final enriched ODNs are sequenced, characterized, and cloned to identify the aptamers with the desired properties [12]. Leveraging their unique three-dimensional folding patterns and molecular recognition capabilities, aptamers can be systematically generated against a broad spectrum of analytes, ranging from simple metal ions and small organic molecules to complex biological entities, including proteins, viruses, bacteria, and even whole cells [13].

Aptamers are widely used in various fields of molecular biology, biotechnology, and biomedical sciences, particularly in gene therapy and targeted therapeutics [14,15,16,17]. Aptamer-based therapies primarily function through two mechanisms: (1) Direct target modulation: Aptamers act as antagonists or agonists to block or activate disease-related molecules. A prominent example is Pegaptanib, the first clinically approved aptamer drug, which selectively binds and neutralizes the vascular endothelial growth factor A165 (VEGF-A165), thereby suppressing pathological angiogenesis and vascular hyperpermeability in conditions like wet age-related macular degeneration [18]. (2) Therapeutic delivery: Aptamers serve as carriers to transport agents (e.g., siRNA, miRNA, chemotherapeutics, or toxins) to specific cells or tissues, enhancing local efficacy while minimizing systemic toxicity. Beyond these roles, aptamers can functionalize nanomaterials (e.g., nanoparticles, liposomes, polymeric micelles, or DNA nanostructures) to enable targeted drug delivery [19]. While existing reviews comprehensively cover aptamer-nanomaterial systems, this review focuses on aptamer–ODN chimeras, excluding detailed discussions of nanomaterial-based approaches [20,21,22,23,24].

### 2.2. The Internalizing Capacity of Aptamers

The internalization of aptamers is particularly important for in vivo applications in targeted delivery [25]. The vast majority of aptamers (such as the transferrin receptor (TfR) aptamer and protein tyrosine kinase 7 (PTK7) aptamer) enter cells through receptor-mediated endocytosis (RME). The specific mechanism of this process is as follows: The aptamer binds to specific receptors on the cell surface to form a complex. The complex then undergoes ligand-receptor clustering on the membrane surface, thereby triggering clathrin-mediated endocytosis (CME) or caveolae-mediated endocytosis (CvME) to internalize the complex into the cell. Notably, a small number of aptamers (e.g., nucleolin aptamers) can achieve receptor-independent internalization through macropinocytosis [25,26]. Afterwards, the aptamers either escape from the endosomes or are transported to lysosomes for degradation. Further studies have revealed that the cellular uptake pathway of aptamer-drug conjugates (ApDCs) is identical to that of aptamers. The aptamer directs ApDC to specific endocytic pathways, while the small-molecule drug does not interfere with their targeting or endocytosis. This confirms that the aptamer indeed plays a dominant role in the internalization of ApDCs [27].

There are two common ways to obtain cell-internalizing aptamers, one involves generating aptamers against predefined cell membrane protein targets; while the other, known as cell-internalization SELEX (also referred to as cell-SELEX or whole-cell SELEX or WC-SELEX), enables the generation of aptamers for target cells without the need to identify the target protein prior to the SELEX process [28,29]. WC-SELEX allows aptamers to be generated in their native environment even when structural information about the target is not yet known. In addition, by tuning the SELEX method, aptamers with unique intracellular behaviors can be developed [30]. Aptamers selected through both methods, targeting membrane proteins with internalizing properties, are being utilized to develop targeted delivery systems by conjugating them to therapeutics such as ODNs and toxic drugs.

### 2.3. Aptamers as Vehicles for Targeted Delivery of ODN

Targeted drug delivery necessitates precise therapeutic agent transport to specific sites/cells while minimizing off-target toxicity. Aptamers serve as ideal targeting ligands for this purpose. Compared with traditional targeting ligands such as antibodies, aptamers offer the following unique advantages: (1) a compact size that enables deeper tissue penetration; (2) exceptional thermal/chemical stability which supports long-term storage and functional recovery post-denaturation; (3) cell-free automated synthesis that ensures precision, reproducibility, and scalability; (4) flexible chemical modifications which enhance stability and enable conjugation with diverse materials; and (5) low immunogenicity/toxicity due to biocompatibility. In contrast, antibodies face the following limitations: temperature-sensitive inactivation, complex/costly live-cell production (risk of contamination), conformational disruption upon modification, poor tissue permeability due to a large size, and immunogenicity (e.g., ADA responses with murine/chimeric forms) [31,32,33,34]. With the above advantages, aptamers are increasingly used in targeted drug delivery.

Leveraging their unique advantages, aptamers can be programmably conjugated with diverse ODNs via molecular engineering, emerging as a core technology for ODN-target delivery. Compared with traditional ODN delivery systems (e.g., lipid nanoparticles or polymeric nanoparticles), aptamer–ODN chimeras exhibit the following features: (1) precise targeting: aptamers directly bind target cells via their intrinsic 3D structures, without requiring surface modifications like other systems; (2) high safety: Composed entirely of nucleic acids, aptamer–ODN chimeras show minimal immunogenicity, making them suitable for long-term or repeated administration, avoiding immune activation or the long-term accumulation risks associated with conventional carriers; (3) enhanced tissue penetration: their small size facilitates infiltration into dense tissues (e.g., solid tumors), while rapid renal clearance reduces systemic toxicity; and (4) scalable production: solid-phase synthesis ensures batch-to-batch consistency, with lower costs and complexity compared to nanocarrier fabrication. While aptamer–ODN chimeras also face limitations, such as susceptibility to nuclease degradation and difficulty in escaping from endosomes [35].

## 3. Aptamer–ODN Chimeras

Following the landmark breakthrough by Giangrande, PH’s team in designing the first aptamer-siRNA chimera [36], research on aptamer–ODN chimeras has expanded significantly. The types of ODNs delivered by aptamers have diversified to include siRNA, ASO, miRNA, and others. These ODNs can flexibly target the DNA or mRNA of key signaling pathway proteins (e.g., STAT3, Bcl-2), which are critical to disease-related processes such as signal transduction, cell proliferation, immune regulation, and apoptosis. Concurrently, the diversity of aptamer targets has rapidly increased, with common binding sites including disease-associated cell surface membrane proteins such as PSMA, HIV-1 gp120, EpCAM, CD4, HER2, EGFR, and nucleolin [37,38,39,40,41,42]. This broad target spectrum endows aptamer–ODN delivery systems with wide therapeutic applicability.

The combinatorial strategies of aptamer–ODN chimeras have evolved from simple covalent linkages to more sophisticated designs, including sticky bridge ligation, chemical crosslinker-mediated conjugation, and modular assembly systems [43]. Covalent linkage refers to the connection of two ODN chains using a phosphodiester bond, in which case the aptamer–ODN chimeras can be synthesized by solid-phase synthesis. Sticky bridge ligation utilizes designed complementary sequences (“sticky bridges”) to form hybrid structures that guide the ligation of two short nucleic acid strands. Chemical crosslinker-mediated conjugation employs bifunctional chemical reagents (e.g., glutaraldehyde, DSP) to directly form covalent bonds between molecules. This method enables the conjugation of nucleotides with other molecules, such as proteins. Modular assembly systems treat nucleic acid molecules (such as aptamers and ODNs) as independent functional modules, which can be combined in a controlled and flexible manner through predefined interfaces and connection strategies. This approach emphasizes module standardization and diversified connection strategies, enabling different functional components to be freely assembled like building blocks, thereby facilitating the rapid construction of chimeras tailored to specific functional requirements. Different connection strategies can be selected according to design requirements. These evolving conjugation strategies have significantly advanced the development of aptamer–ODN chimeras for therapeutic applications.

### 3.1. Aptamer-siRNA Chimeras

Small interfering RNA (siRNA) represents a class of short synthetic RNA duplexes, typically consisting of 21–23 nucleotide base pairs, with each strand terminating in characteristic 2-nucleotide 3′ overhangs. The mechanism of action involves the recruitment of siRNA duplexes into the RNA-induced silencing complex (RISC). An RISC is composed of multiple proteins and a small RNA molecule (e.g., siRNA, miRNA). One of the core components is the Argonaute protein, especially Ago2 (Argonaute 2), which is a key enzyme in RISC and has endonuclease activity that can catalyze the cutting of target mRNA. Within the RISC machinery, ATP-dependent strand separation occurs, resulting in the selective retention of the antisense (guide) strand within the activated RISC. The guide strand is complementary to the target mRNA, facilitating a sequence-specific recognition of complementary mRNA targets through Watson–Crick base pairing. Subsequent endonucleolytic cleavage, mediated by the PIWI domain of the Ago2 catalytic subunit, disrupts mRNA integrity and abrogates translational elongation [44,45,46,47]. siRNA exhibits exceptional therapeutic potential due to its high target gene specificity, relatively low immunogenicity, and straightforward design and validation processes [48]. Despite the therapeutic potential of siRNA, its clinical translation is hindered by delivery challenges, including rapid degradation in circulation and inefficient intracellular delivery. Consequently, designing targeted and stable delivery systems is essential. The covalent conjugation of aptamers with siRNA has emerged as a rational delivery paradigm. This approach offers advantages such as enhanced cellular uptake efficiency, improved cell-type specificity through aptamer-mediated targeting, and a reduced reliance on additional delivery vehicles, thereby improving the tolerability and safety profile of the delivery formulation [49,50]. Table 1 summarizes the progress in aptamer-siRNA chimera therapy.

The team led by Giangrande, PH, developed the first aptamer-siRNA chimera capable of delivering functional siRNA to specific cell types. This system combined a prostate-specific membrane antigen (PSMA) aptamer with siRNA targeting the mRNA of polo-like kinase 1 (Plk1) or B-cell lymphoma-2 (Bcl-2). PSMA is a transmembrane receptor that is highly expressed on prostate cancer cells and tumor-associated vascular endothelium, particularly in advanced metastatic castration-resistant prostate cancer (mCRPC), and its expression in other normal tissues is minimal. Therefore, it is an ideal therapeutic target in the treatment of prostate cancer [108]. Plk1 is a serine/threonine protein kinase that plays a central role in cell cycle regulation and is frequently overexpressed in tumor cells, making it a key therapeutic target in cancer [109]. Bcl-2, critical for regulating mitochondrial apoptosis pathways, inhibits programmed cell death. Its overexpression in certain cancers enables tumor cells to evade apoptosis and survive persistently, establishing it as a prominent target for anticancer therapies [110]. The PSMA aptamer-siRNA chimera bound with high affinity to PSMA through specific molecular recognition. Following RME, the chimera entered the target cells. Subsequently, the siRNA component underwent Dicer-mediated processing, ultimately leading to the degradation of Plk1- and Bcl-2-encoding mRNAs. By modulating both cell cycle progression and the apoptosis pathways, this chimera demonstrated potent therapeutic effects, including significant tumor growth suppression and observable phenotypic regression. The team further optimized the PSMA aptamer-siRNA chimera through chemical modifications, enhancing its silencing efficacy and specificity. In this design, the aptamer can be replaced with specific sequences targeting other tumor biomarkers (e.g., EGFR, HER2), while the siRNA can be customized to target the key mRNAs associated with various diseases, thereby providing a foundational framework for personalized treatment.

**Figure 2:**
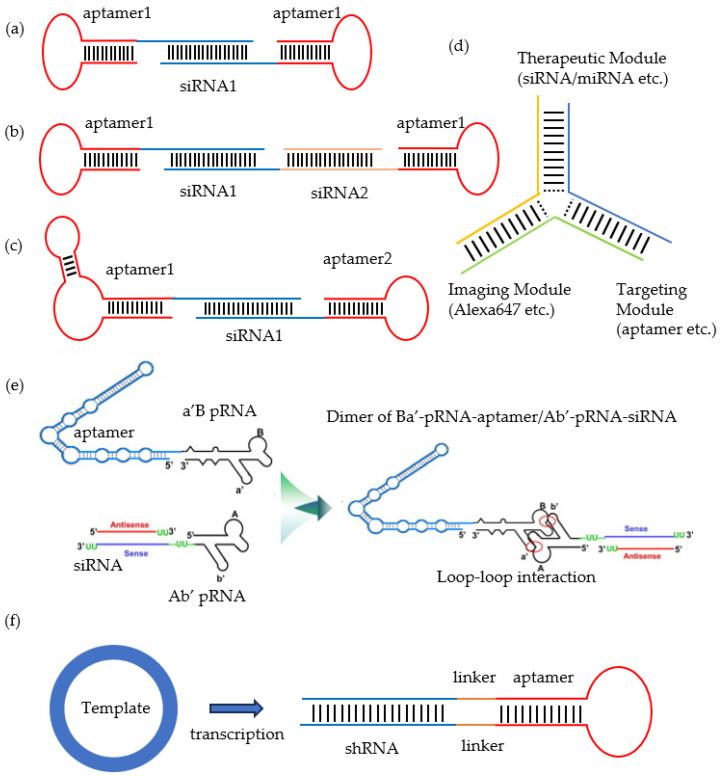
Construction methods of aptamer-siRNA chimeras. (**a**) Chimera of bivalent aptamers and siRNA. (**b**) Chimera of bivalent aptamers and two different siRNAs. (**c**) Chimera of heterobivalent aptamers and siRNA. (**d**) The 3WJ-RNA nanoparticle structure connects three different modules: a therapeutic module, an imaging module, and a targeting module. (**e**) Aptamer-siRNA chimera using pRNA as a linker, the red circle indicates the interlocking interaction between the left and right loops of pRNA. (**f**) Long-term expression of shRNA and aptamers using expression vector. Figure 2e is reproduced with permission from Ref. [63].

To enhance the target recognition capability of aptamers, multivalent aptamers were employed to construct aptamer-siRNA chimeras. For example, Barth, S et al. designed a bivalent aptamer-siRNA chimera by attaching the sense strand of the siRNA to the 3′ end of the first aptamer and the antisense strand to the 3′ end of the second aptamer. This configuration generated a precisely spaced 21-nucleotide double-stranded RNA (dsRNA) junction region (Figure 2a). Experimental validation revealed that the bivalent chimera exhibited both enhanced target affinity (while maintaining aptamer specificity) and a fourfold increase in internalization efficiency compared to the monovalent counterpart [111]. Subsequent studies utilizing similar bivalent architectures for siRNA delivery have reported comparable efficacy improvements [86,112].

Treatment approaches using chimeras to deliver two or more different siRNAs for combined therapy have also been developed. Liu, HY et al. developed a bivalent aptamer-dual siRNA chimera, which consists of a bivalent PSMA-specific aptamer and two distinct siRNA sequences targeting independent oncogenic pathways (EGFR and Survivin), along with a tetra-uracil (UUUU) spacer that serves as a functional linker to separate the siRNA duplexes (Figure 2b) [56]. Experimental validation demonstrated robust tumor-targeted delivery and the potent gene silencing efficacy of the EGFR- and survivin-targeting siRNAs, resulting in a significant reduction in tumor size and the inhibition of tumor-associated vasculature. The innovative bivalent aptamer-dual siRNA chimera strategy has opened new avenues for tumor-targeted therapy. Traditional single-targeted therapies may encounter challenges such as target escape and the development of drug resistance, whereas combined therapies can simultaneously target multiple carcinogenic pathways, thereby enhancing efficacy and reducing the risk of drug resistance.

Heterodimeric aptamer-siRNA chimeras were developed to address the problem of single-target resistance. The HER family comprises four functionally interdependent and compensatory members: the epidermal growth factor receptors (EGFR/HER1), HER2, HER3, and HER4 [113]. These receptors are closely related to tumor cell proliferation, invasion, and drug resistance and are overexpressed in tumor cells. Previous studies have demonstrated that antibody-based combinatorial targeting of EGFR, HER2, and HER3 achieves significantly higher efficacy than single-target approaches [114]. Inspired by this evidence, Liu, HY’s team, innovatively engineered a heterodimeric aptamer-siRNA chimera incorporating 2–4 unpaired nucleotide spacers between each aptamer and siRNA component (Figure 2c) [85]. This chimera integrates HER2- and HER3-specific aptamers with double-stranded EGFR-targeting siRNA, enabling each aptamer to perform dual roles: (1) acting as a receptor antagonist to inhibit ligand binding, and (2) serving as a cell-type-specific delivery vehicle for siRNA. Experimental validation revealed that the chimera simultaneously downregulated all three receptor expression levels, consequently inducing apoptosis. This innovative three-in-one design provides a novel therapeutic strategy to address the complexity of the HER family protein network, and the challenges associated with single-target therapy resistance. It highlights the significant potential of multi-targeted therapy in tumor immunotherapy, particularly for treating drug-resistant tumors and tumors activated by multi-receptors.

The three-way junction (3WJ) motif derived from the phi29 DNA packaging motor’s packaging RNA (pRNA) has been extensively used to link aptamers with diverse ODNs [57,82,83,88,115,116,117,118], demonstrating significant potential for developing RNA nanoparticles for disease treatment. The 3WJ architecture allows for the flexible modular loading of functional units, including the following: imaging modules (e.g., fluorophores or fluorescent aptamers), targeting modules (e.g., aptamers or chemical ligands), and therapeutic modules (e.g., siRNA, ASO, saRNA, miRNA, anti-miRNA, or chemotherapeutic agents). Each module operates independently while retaining structural and functional integrity (Figure 2d) [119,120]. These nanoparticles not only exhibit targeting and therapeutic effects but are also easy to image and track, providing a solid foundation for the widespread application of 3WJ nanoparticles in biomedical fields. Another chimeric RNA structure engineered from the phi29 DNA packaging motor also serves as a linkage bridge between aptamers and ODNs [63,69,100]. This structure can spontaneously form stable dimers through its unique staggered arrangement of left-hand and right-hand loops. The system consists of two core modules: the pRNA-aptamer module, which is responsible for the specific recognition and binding of cell surface receptors, and the pRNA-ODN module, which facilitates the efficient delivery of ODN (Figure 2e). This bifunctional chimeric system provides a novel technological platform for the development of cell type-specific ODN delivery vectors.

Short hairpin RNAs (shRNA) are synthetic RNA molecules composed of three components: a target-specific region, a spacer region, and a reverse complementary segment of the target sequence [121]. Compared to siRNA, shRNA contains an additional loop structure. After transcription, shRNAs are cleaved into small double-stranded RNA molecules by processing enzymes such as Dicer within the cell, converting them into functional siRNAs that enter the RNA interference (RNAi) pathway to target and degrade specific mRNAs [122]. Dicer is an important enzyme that cleaves long double-stranded RNA or precursor miRNA into small RNA fragments, initiating the gene silencing process. The combination of shRNA and aptamer follows a design concept similar to siRNA-aptamer chimeras. Specifically, these chimeras achieve targeted delivery by linking shRNA with specific receptor aptamer, allowing for the precise delivery of shRNA to particular cells or tissues. The aptamer component enhances the efficient entry of shRNA molecules into target cells by recognizing and binding to specific receptors on the cell surface, while the shRNA inhibits the expression of target mRNAs specifically through RNAi mechanisms within the cells [103,104,105].

Unlike siRNA, shRNA can be stably expressed via expression vectors. Therefore, by incorporating the aptamer sequence into the shRNA expression vector via a rational design, the stable expression of the aptamer-shRNA chimera can be achieved. Rossi, JJ’s team, inserted an anti-HIV integrase aptamer into the loop region of the Tat-Rev shRNA. HIV integrase is a key enzyme in the HIV replication cycle, responsible for integrating viral DNA into the host genome, thereby enabling long-term latency and continuous viral replication [123]. Tat and Rev are two key regulatory proteins in the HIV-1 lifecycle, playing central roles in the transcriptional activation of viral genes and RNA nuclear export, respectively. The design of this expression vector allows for the continuous production of large amounts of aptamer-shRNA chimeras through transcription (Figure 2f), thus achieving the long-term inhibition of HIV replication in cell culture systems [106]. The combination of shRNA and aptamers can be flexibly used to target the same gene (an mRNA and its corresponding protein), two genes (an mRNA and a different protein), or a protein and a non-coding RNA. Moreover, by using multiplexing vectors, multiple shRNA-aptamer chimeras can be expressed from a single transcript, enabling the simultaneous inhibition of multiple targets, which is of significant importance in combating the ever-evolving HIV.

### 3.2. Aptamer-ASO Chimeras

Therapeutic antisense oligonucleotides (ASOs) are synthetic, single-stranded DNA or RNA molecules, typically 18–30 nucleotides in length [124]. Their mechanisms of action vary depending on factors such as the target sequence, chemical composition, and nucleotide type. For example, ASOs can act through RNase H-mediated mRNA degradation, the spatial blockage of translation initiation, or the modulation of splicing patterns [125,126,127]. Depending on the mechanism, ASOs can target coding or non-coding RNAs, increase or decrease RNA or protein levels, as well as mask or disrupt miRNA and protein interactions, offering great flexibility in use [127,128]. ASO therapeutics offer several advantages, including a flexible design architecture that allows for sequence optimization, ease of chemical modification to enhance stability and specificity, scalable and reproducible synthesis protocols, and well-established quality control parameters. However, a major obstacle to clinical translation lies in developing effective delivery systems capable of overcoming biological barriers [129,130,131]. Aptamer–ASO chimeras offer a promising solution by facilitating targeted delivery, reducing the required ASO dosages and associated off-target effects, and improving safety [7]. Table 2 summarizes the research progress in aptamer-ASO chimera therapy.

Aptamer-ASO chimeras exhibit excellent targeted delivery and therapeutic capabilities. Nucleolin is a ubiquitous, multifunctional protein predominantly located in the nucleolus, nucleoplasm, cytoplasm, and cell membrane. It is overexpressed in various cancers, and plays a multifaceted role in tumor initiation and progression [139]. AS1411 is a guanosine-rich ODN aptamer that binds to nucleolin and is subsequently internalized by tumor cells [140]. Kotula, JW et al. designed an aptamer-ASO chimera composed of an AS1411 aptamer targeting nucleolin and a splice-switching oligonucleotide (SSO) targeting luciferase. This chimera demonstrated an approximately 5-fold greater potency than the SSO alone in the test cells, highlighting the therapeutic value and nuclear delivery capacity of aptamer chimeras [132]. Similarly, Hong, SN et al. constructed an AS1411 aptamer-galectin-1 ASO chimera, which also demonstrated improved cellular uptake and selective entry into cells [135]. Tanaka, K et al. employed cell-internalization SELEX technology to screen and modify cell-internalizing aptamers for delivering ASOs into A549 cells, thereby accelerating the use of these aptamers as ODN delivery vehicles [28].

**Figure 3:**
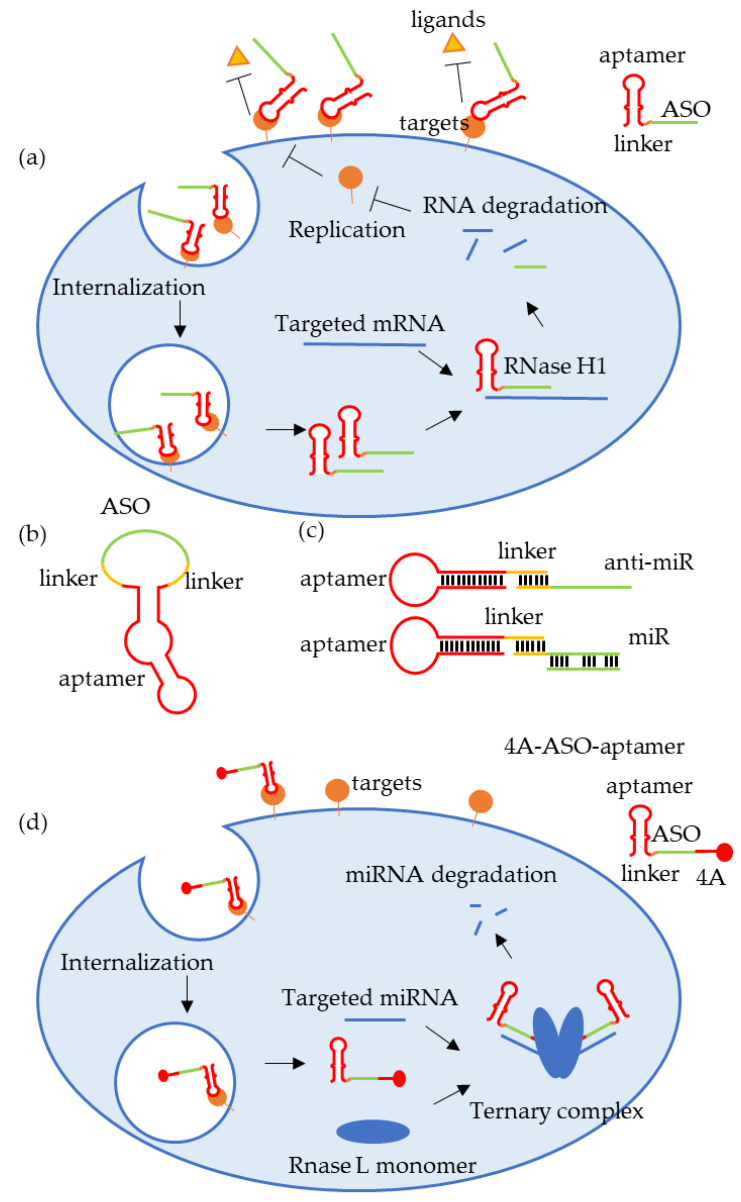
(**a**) The aptamer-ASO chimera delivery system with dual inhibitory functions. (**b**) The circSApt-ASO chimera. (**c**) Aptamers used for delivery of miRNA and anti-miRNA. (**d**) 4A-ASO-aptamer chimera. The aptamer facilitates the entry of 4A-ASO-aptamer into cancer cells, where 4A recruits and activates RNase L to degrade the target miRNA.

As mentioned above, aptamers can function as either agonists or antagonists in therapy, as well as drug delivery vehicles, enabling them to play a dual role in drug delivery [11]. Programmed death-ligand 1 (PD-L1) is an immune-regulatory protein with a complex subcellular distribution and multiple isoforms. Its receptor, PD-1, interacts with PD-L1 to form an immune checkpoint pathway that plays a critical role in tumor immune evasion. Tumor cells or tumor-associated immune cells frequently overexpress PD-L1, which binds to PD-1 on T cells, thereby inhibiting T cell function and evading immune clearance [141]. Current therapeutic strategies mainly target membrane-bound PD-L1 (mPD-L1), but new evidence suggests that intracellular and other forms of PD-L1 also influence anti-cancer immune responses [142]. Luo, FT et al. developed a novel antagonistic aptamer-ASO chimera with dual functionality: PD-L1-specific antagonism mediated by the aptamer domain, and targeted PD-L1 suppression via ASO-mediated gene silencing (Figure 3a) [137]. This bifunctional construct leverages the aptamer’s antagonistic capacity to disrupt PD-L1/PD-1 interactions, while concurrently facilitating the intracellular delivery of the conjugated ASO, enabling comprehensive PD-L1 suppression through both a surface receptor blockade and total protein downregulation. This strategy enhances immune activation compared to single-mechanism inhibitors and amplifies therapeutic outcomes through the synergistic effect of a checkpoint blockade and gene silencing, representing a promising and broadly applicable ODN-based immunotherapy strategy.

In addition to targeted delivery, a major challenge in ODN therapeutics is their inherent susceptibility to nuclease-mediated degradation. To address this limitation, Yang, G et al. pioneered the development of a circularized aptamer-ASO chimera designed for the dual suppression of SARS-CoV-2 viral proliferation and the associated inflammatory responses [138]. This innovative construct consists of three functionally integrated components: (1) A spike-targeting aptamer (SApt): a high-affinity ligand that specifically recognizes the trimeric spike protein of severe acute respiratory syndrome coronavirus 2 (SARS-CoV-2). It blocks viral entry by competitively inhibiting the spike protein–ACE2 receptor interaction and serves as a targeting moiety for delivering the chimera to infected cells. (2) A nucleocapsid-specific ASO (NASO): a therapeutic ODN engineered to silence the SARS-CoV-2 nucleocapsid (N) gene, thereby mitigating the N protein-mediated inflammatory cascades. (3) An optimized linker sequence: a structural element that preserves molecular flexibility and ensures the functional accessibility of the aptamer and ASO domains (Figure 3b). The circularized aptamer-ASO chimera significantly increases the stability of ODN in serum, establishing a novel strategy for ASO stabilization. This design overcomes the intrinsic limitations of linear architectures, and enables multifunctional integration within single-molecule entities, providing forward-looking strategies for molecular medicine.

### 3.3. Aptamer-miRNA Chimeras

As highly conserved endogenous regulatory molecules, microRNAs (miRNAs) are a class of non-coding RNAs (ncRNAs), typically 22 nucleotides in length. They mediate post-transcriptional gene silencing through the formation of incomplete base-pairing between their 5′-end seed region (nucleotides 2–8) and the 3′-untranslated region (3′-UTR) of target mRNAs [143]. This sequence-specific regulatory mechanism enables miRNAs to simultaneously regulate the expression networks of hundreds of target genes, playing a central role in biological processes such as cell cycle progression, proliferation, differentiation, metabolic homeostasis, and programmed cell death. Consequently, miRNAs are regarded as novel therapeutic targets with significant potential [144]. Given the abnormal expression profiles of miRNA in various pathological states, miRNA therapies can take the form of mimics, inhibitors, or combinations with other drugs. Mimic therapies involve introducing synthetic miRNAs to complement low-expressed functional miRNAs. These mimics re-suppress their target genes and subsequently normalize cellular processes to inhibit disease progression. In contrast, miRNA inhibitory therapies include the introduction of anti-miRNA ODNs (anti-miRs), miRNA masking, and miRNA sponges. Anti-miRNA ODNs (AMOs) have sequences complementary to the target miRNA, thus preventing the miRNA from binding to its mRNA target [145].

Achieving the efficient delivery of miRNA-based therapies remains a key bottleneck in clinical translation. Aptamers provide an innovative solution to overcome this limitation. The design methods for the aptamer-based delivery of miRNAs and anti-miRs involve ligating aptamers to miRNAs or anti-miRs using strategies such as sticky bridge technology or chemical crosslinking (Figure 3c). These aptamers can be rapidly internalized by cells expressing their specific targets, providing an effective tool for delivering miRNAs and anti-miRs [115,116,117,146,147,148,149,150,151,152,153,154,155,156,157,158,159] (Table 3).

MiRNA degradation is also an effective strategy of miRNA therapy. RNA-inducible ribonuclease-targeted chimeras (RIBOTACs) are designed to achieve precise RNA degradation inspired by the concept of protein degradation-targeted chimeras (PROTACs) [160,161]. Their core molecular architecture consists of two functional domains: an RNA-binding domain, which achieves the specific recognition of target RNAs via ASOs or small molecule ligands; and an RNase L-recruiting domain, which recruits and activates ribonuclease to cleave the RNA, leading to its degradation [160]. RNase L recruiters can be either four 2′-5′-linked oligoadenylate units (4A), which are naturally present in cells, or small molecules obtained through DNA-encoded library screening. Building on this technology, Fang, Y et al. pioneered the aptamer-RIBOTAC (ARIBOTAC) platform by designing a 4A-ASO-AS chimera composed of three parts: (1) an intermediate ASO that exclusively recognizes the target miRNAs; (2) a 5′-end of the ASO coupled with the 4A structure that recruits and activates endogenous RNase L via monomer dimerization; and (3) a 3′-end AS1411 aptamer for targeting tumors and facilitating intracellular delivery. The 4A-ASO-AS chimera is specifically internalized into cancer cells overexpressing nucleolin via AS1411, followed by the recruitment and activation of RNase L by the 4A structure and miRNA recognition by the ASO. The formation of this ternary complex narrows the distance between the miRNA and RNase L, allowing the conserved motifs of the target miRNA to be localized in the catalytic center of RNase L. This mechanism enables site-specific cleavage and demonstrates a stronger antitumor effect than traditional miRNA inhibitors in murine models (Figure 3d) [162]. The modular design of ARIBOTAC technology offers a high degree of programmability: (1) different miRNAs (e.g., let-7 family or miR-155) can be targeted by replacing the ASO sequence; (2) the aptamer element can be substituted with other tumor-specific ligands such as EpCAM or PSMA; and (3) the entire structure, including the 2′–5′A activation module, can be optimized to enhance nuclease resistance. This flexible architecture provides a molecular basis for the development of a “universal” RNA degradation platform, which holds great translational value for the treatment of miRNA dysregulation-associated tumors and RNA virus infections.

### 3.4. Aptamer-saRNA Chimeras

Small activating RNAs (saRNAs) share high structural homology with siRNAs, consisting of 21 base pairs of double-stranded RNA with a characteristic 3′ overhang at the 3′ end. However, these two types of non-coding RNAs exhibit opposing effects on gene regulation [163]. Unlike siRNAs, which silence gene expression, saRNAs enhance the expression of target genes, leading to transcription levels that exceed basal expression. This unique gene-activating property makes saRNAs valuable in translational medicine, particularly in the field of therapeutic gene upregulation [164]. Current clinical studies on saRNAs primarily focus on the transcriptional activation of the *CCAAT/Enhancer Binding Protein Alpha (CEBPA*, *or C/EBPα)* gene for the treatment of leukemia and solid tumors [165]. CEBPA is a transcription factor involved in regulating various cellular processes, and its downregulation is strongly associated with the pathology of many malignant tumors. MiNA Therapeutics (a company) developed CEBPA-51 saRNA to specifically upregulate the transcription of the *CEBPA* gene and restore its expression [166]. This saRNA has demonstrated anti-tumor efficacy in different preclinical models using various delivery systems. Table 4 summarizes the research progress in aptamer-saRNA/decoy chimera therapy.

Rossi, JJ et al. conjugated *CEBPA*-saRNA molecules with different aptamers to construct two composite delivery systems that specifically recognize pancreatic ductal adenocarcinoma (PDAC). They coupled *CEBPA*-saRNA with either a PDAC-specific aptamer or an anti-human transferrin receptor (hTfR) aptamer. The PDAC-specific aptamer was screened and optimized using whole-cell SELEX to ensure specific recognition of cell surface epitopes. hTfR is a type II transmembrane glycoprotein receptor that mediates the endocytosis and transport of trivalent ferric ions. Its expression can be upregulated nearly 100-fold in malignant, proliferating cell populations, making it an ideal molecular target for tumor-targeted therapy [173]. Studies have demonstrated that the targeted delivery of *CEBPA*-saRNA using either anti-hTfR or PDAC-specific aptamers leads to efficient endocytosed by pancreatic cells, resulting in a significant increase in *CEBPA* transcript and protein levels, as well as a notable tumor-suppressive effect [167,168,174].

### 3.5. Aptamer-Decoy Chimeras

Decoy technology includes two main approaches, decoy ODNs and decoy peptides [175]. Decoy ODNs are short, double-stranded molecules that mimic the DNA binding sites of the gene promoters and compete with native DNA sequences for transcription factor binding, thereby altering the expression of all downstream genes regulated by those transcription factors. This mechanism holds significant potential for regulating the transcription of disease-associated genes [176]. However, compared to ASO or siRNA methods, decoy ODNs are less specific in their mechanism of action. Their lack of tissue and cell specificity, along with susceptibility to degradation during endocytosis, limits their applications. Therefore, it is necessary to establish an effective delivery mechanism to transport decoys to the desired site of action [177]. In this context, aptamers can be used to facilitate the targeted delivery of decoys.

**Figure 4:**
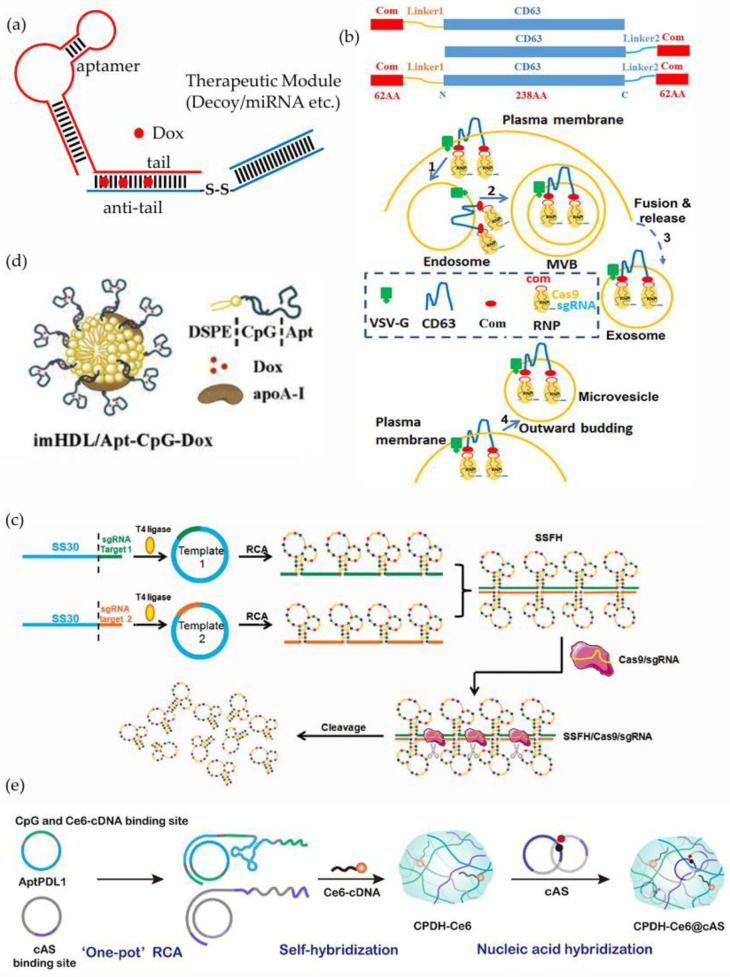
(**a**) Schematic illustration of DOX loading into aptamer–ODN chimeras. (**b**) Schematic illustration of enriching RNPs in EVs. Reproduced with permission from Ref. [179]. (**c**) Schematic illustration of a novel CD123 polyaptamer hydrogel edited by Cas9/sgRNA, reproduced with permission from Ref. [180]. (**d**) Schematic illustration of the immune lipoprotein imHDL/Apt-CpG-Dox, reproduced with permission from Ref. [181]. (**e**) Schematic illustration of an embedded photoresponsive immunotherapy hydrogel, reproduced with permission from Ref. [182].

The double-stranded region in the middle of the aptamer–ODN chimera can be used to load with chemotherapeutic agents such as doxorubicin (Dox) to achieve synergistic therapy. Porciani, D et al. designed an aptamer-decoy chimera (aptacoy), consisting of an aptamer targeting the transferrin receptor (TfR) and a nuclear factor κB (NFκB) decoy. The two strands are assembled into an overall structure through complementary pairing at their ends, and Dox is loaded into the double-stranded DNA structure of aptacoy (Figure 4a) [171]. TfR is overexpressed in many solid tumors, while NF-κB is a ubiquitous transcription factor that suppresses Dox-induced apoptosis [178]. The experimental results showed that the aptamer motif facilitates the efficient and selective delivery of therapeutic molecules to tumor cells, while the combination of Dox and the NF-κB decoy enhances cellular sensitivity to DOX and amplifies its pro-apoptotic effects. The design of aptacoy aims to address the key challenges of poor selectivity and drug resistance associated with traditional chemotherapeutic drugs through the three-pronged strategy of “targeted delivery + drug loading + signaling pathway intervention”.

### 3.6. Aptamer-sgRNA Chimeras

CRISPR-Cas9 is a gene-editing technology capable of making precise cuts and modifications at specific locations in DNA molecules. As a representative of the third generation of gene editing technology, the CRISPR-Cas9 system has triggered a paradigm shift in the field of molecular genetics due to its programmability, efficiency, and precision [183]. The core components of this system include the Cas9 nuclease and single-guide RNA (sgRNA), which form an RNA-DNA heteroduplex with the target DNA via a spacer sequence at the 5′ end of the sgRNA. Cas9 functions as a molecular scissor, cutting the DNA at the target site, and resulting in double-stranded DNA breaks (DSBs). Subsequently, the cellular repair mechanism introduces modifications at the target site through non-homologous end-joining (NHEJ) or homology-directed repair (HDR) [184,185]. In clinical applications, the safe and effective delivery of CRISPR/Cas9 remains a critical challenge. Delivery strategies can be categorized into three major types based on their molecular format: (1) a plasmid DNA (pDNA) vector system, which involves circular plasmids that can encode the Cas9 and sgRNA; (2) mRNA delivery systems that can express both Cas9 and sgRNA; and (3) the direct delivery of Cas9 and sgRNA as a ribonucleoprotein (RNP) complex. Each approach has its own advantages and disadvantages, as well as specific challenges that must be addressed [186].

Extracellular vesicles (EVs) are emerging as promising tools for delivering RNP complex. However, the lack of an efficient enrichment mechanism for loading RNPs into EVs limits their utility as delivery vehicles [187]. Previous studies have shown that the com (the aptamer named com)/ABP Com (the aptamer-binding protein named Com) pair can package Cas9 and adenine base editor RNPs into lentiviral capsids [188]. Based on this, Yao, XG et al. developed an aptamer-mediated method for RNP loading that enhances the incorporation of RNPs into EVs by leveraging specific interactions between com (inserted into the sgRNA) and Com (fused to CD63). In this technology system, the aptamer com element is assembled into a RNP complex with the Cas9 protein by inserting into the sgRNA backbone. As a signature membrane protein of exosomes, the tetraspanin CD63 has a topological structure with a transmembrane domain that penetrates the exosome membrane, with the N-terminus and C-terminus both located on the cytoplasmic side, and the hydrophilic ring structure extending into the exosome cavity [189]. To achieve precise positioning, the ABP Com was fused to the N-terminus, C-terminus, or both ends of CD63 to ensure that the Com functional domain was stably positioned at the cytoplasmic interface of the plasma membrane. To further improve the efficiency of exosome delivery, the research system co-expressed vesicular stomatitis virus G glycoprotein (VSV-G), which facilitates exosomal escape from endosomes in recipient cells via its membrane fusion capabilities. During exosome generation, the entire RNP complex is enriched in exosomes through interactions involving CD63-Com, com-Com, com-sgRNA, and Cas9. Notably, this enrichment mechanism has pathway diversity; in addition to the classical exosome pathway, RNPs can also be encapsulated in microvesicles formed by budding from the plasma membrane (Figure 4b) [179]. A quantitative analysis showed that this targeted enrichment system could increase gene editing activity by more than ten times compared with the control exosomes lacking an aptamer com or ABP Com. This innovative design not only provides an efficient solution for gene editing delivery, but also establishes a generalizable technical paradigm for engineering modifications in EVs.

In the coupling of aptamers and ODNs, the aptamers do not always function solely as targeting ligands [190]. Wu, HB et al. designed a DNA hydrogel composed of the CD123-targeting aptamer SS30 using rolled-loop amplification (RCA) methods, referred to as the SS30 polyaptamer hydrogel (SSFH). As a therapeutic aptamer, SS30 effectively inhibits the proliferation of acute myeloid leukemia (AML) cells by blocking the IL-3/CD123 signaling pathway, showing significant anti-tumor potential [191,192]. The research team designed two functionalized RCA templates: Template 1 contained the SS30 sequence and the sgRNA target sequence 1, while Template 2 incorporated the SS30 and the sgRNA target sequence 2 complementary to target sequence 1. After amplification, the complementary regions of the two templates enabled specific hybridization, forming a three-dimensional SSFH hydrogel. During the treatment implementation phase, when the SSFH and Cas9/sgRNA complex were co-injected into the target site, the Cas9 precisely cut the hydrogel skeleton by recognizing the specific site guided by the sgRNA, thereby continuously releasing the active SS30 aptamer in a controlled manner (Figure 4c). This design achieves the intelligent release of aptamer through a DNA hydrogel system regulated by CRISPR-Cas9. It not only provides a new strategy for the treatment of AML with high efficiency and low toxicity, but also redefines the role of aptamers and CRISPR tools in therapeutic delivery systems. The coupling of aptamers and sgRNAs also holds great potential in other applications [193,194,195] (Table 5).

### 3.7. Aptamer-CpG Chimeras

CpG ODNs are synthetic single-stranded DNA molecules characterized by a specific sequence (CpG motif), which consists of tandemly linked unmethylated cytosine (C) and guanine (G) connected by phosphodiester bonds. By mimicking the naturally occurring CpG motifs in pathogen DNA, these molecules can specifically activate Toll-like receptor 9 (TLR9) in immune cells, triggering MyD88-dependent signaling pathways that induce the secretion of a variety of cytokines, thereby enhancing the innate immune response and promoting the activation of adaptive immunity. Due to this mechanism, CpG ODNs have been widely used in tumor immunotherapy, anti-infective vaccine adjuvant and allergic disease treatment [208,209]. Despite preclinical studies confirming their significant immune-activating potential, the clinical application of CpG ODNs is hindered by several limitations, including difficult cellular uptake, poor stability, rapid degradation, low targeting efficiency, a short half-life, and insufficient tissue penetration. Therefore, effective delivery strategies must be explored to maximize their therapeutic efficacy and accelerate their clinical translation [210,211]. One promising approach involves the combination of CpG ODNs with aptamers.

Precision delivery and intelligent controlled release are important research directions for improving drug efficacy. Han, Y et al. designed an immunoglobulin nanomedicine named the imHDL/Apt-CpG-Dox composite system. In this design, the AS1411 aptamer was fused with a CpG motif to create an Apt-CpG sequence. This Apt-CpG was then conjugated to the synthetic phospholipid DSPE(1,2-distearoyl-sn-glycero-3-phospho-ethanolamine) to form an Apt-CpG-DSPE molecule, which was subsequently incorporated into the lipid monolayers of high-density lipoprotein (HDL) to construct imHDL/Apt-CpG nanoparticles. HDL is commonly used for drug delivery due to its customizable structure and high biocompatibility. Finally, Dox was loaded into the double-stranded ODN region between the CG base pairs, yielding the complete imHDL/Apt-CpG-Dox composite system (Figure 4d) [181]. Apolipoprotein AI (Apo-AI), the main component of HDL cholesterol, is the ligand for the scavenger receptor class B type I (SRBI), which is expressed at higher levels in many tumor cells and serves as a potential biomarker for tumor diagnosis and prognosis [212]. Once Apo-AI is recognized by the SR-BI receptors at the tumor site, the HDL nanostructures trigger extracellular dissociation, releasing Apt-CpG-Dox into the tumor cell mesenchyme. The AS1411 aptamer in Apt-CpG-Dox further binds to the membrane nucleolin and translocates to the nucleus, where Dox is released and exerts its therapeutic effects. Meanwhile, the CpG motifs induce antigen recognition, pro-inflammatory cytokine secretion, and host anti-tumor immunity. Ultimately, the synergistic action of these components results in an enhanced anti-tumor effect. This design builds an efficient and safe multifunctional nanodrug platform by integrating the targeting capabilities of natural HDL carriers, the membrane and nuclear localization capabilities of AS1411, the immunostimulatory effect of CpG, and the chemotherapeutic effect of Dox. Its core advantage lies in maximizing therapeutic efficacy while minimizing side effects through precise delivery and intelligent controlled release, providing an innovative strategy for tumor treatment.

Wang, DY et al. developed an intelligent DNA hydrogel system that integrates tumor recurrence monitoring and photodynamic immune regulation. The system is based on two functionalized circular DNA templates: one encodes the PD-L1 aptamer and the CpG sequence, while the other encodes the tumor recurrence sensor binding domain. These templates are co-assembled with the photosensitizer Ce6-cDNA through a one-step rolling circle amplification (RCA) process. Further, a circular ATP sensor (cAS) is incorporated into the hydrogel network through a sequence complementation strategy to construct a CPDH-Ce6@cAS composite system (Figure 4e) [182]. In the clinical translation design, the hydrogel can be implanted in situ in the surgical area after tumor resection. The PD-L1 aptamer selectively captures early recurrent tumor cells, and the resulting cell aggregation effect significantly increases the local ATP concentration. The cAS component responds to the ATP gradient, activating a fluorescence signal to enable real-time monitoring of tumor recurrence. Upon detection of a positive signal, near-infrared (NIR) laser irradiation activates the Ce6-mediated photodynamic effect. The generated reactive oxygen species (ROS) not only directly kill tumor cells but also trigger the disassembly of the hydrogel network, thereby accurately releasing the PD-L1 aptamer and CpG sequence. The released PD-L1 aptamer blocks the PD-1/PD-L1 immune checkpoint, while the CpG motif stimulates the dendritic cell-T cell immune cascade reaction, ultimately achieving synergistic immunotherapy. This design integrates diagnosis, treatment, and immune regulation functions into a single platform through precise programming at the molecular level, realizing the closed-loop management of “monitoring–warning–treatment” for postoperative recurrence, providing an innovative strategy for the postoperative treatment of tumors, and demonstrating the application potential of precision medicine in the field of integrated molecular diagnosis and treatment.

## 4. Conclusions

Since the discovery of aptamers in the 1990s, their applications in the biomedical field have gradually expanded, especially in targeted drug delivery systems [213]. With the development of ODN-based therapies, aptamers have been extensively explored for binding to various types of ODNs (such as siRNA, miRNA, ASO, etc.) to form aptamer–ODN chimeras. These chimeras not only facilitate targeted delivery, but also enable the release of ODNs following cellular internalization, thereby achieving therapeutic purposes such as gene silencing [214]. At present, aptamer–ODN chimeras have shown promise in various research fields, especially in cancer, viral infections, and genetic diseases [35,215]. Despite their broad potential, the widespread application of aptamer–ODN chimeras in the biomedical field still faces several challenges. First, the challenge of ODN release: even after successful internalization, the chimeras must escape from endosomes/lysosomes to release the ODNs, and cellular uptake efficiency remains difficult to control. Second, the challenge of nucleic acid degradation: both aptamers and ODNs are nucleic acid molecules and are susceptible to degradation by nucleases. Moreover, extensive preclinical studies and clinical trials are necessary to ensure the safety and efficacy of these chimeras in humans. Finally, as a novel therapeutic strategy, aptamer–ODN chimeras may encounter complex regulatory approval processes, and determining how to meet regulatory requirements represents an additional hurdle.

In order to expand its application in biomedicine and related fields, the research on aptamer–ODN chimeras can focus on the following directions: (1) The optimization of design strategies: a. The enhancement of molecular stability: Aptamers and ODNs are easily degraded by nucleases in vivo and typically exhibit short half-lives. To improve their stability, chemical modifications are necessary. They may include thiophosphate bonds (PS backbone), 2′-O-methylation (2′-OMe), and locked nucleic acids (LNA) to enhance resistance to nucleases such as RNase H and serum nucleases. The aptamer backbone and ODN bases can be optimized separately to avoid mutual interference [32]. Moreover, circular aptamer–ODN structures can significantly improve stability by eliminating nuclease recognition sites, thus enhancing resistance to degradation [138]. b. The design of precision targeting systems: creating precise targeting systems that allow aptamers to recognize multiple distinct cell surface receptors or multiple copies of the same receptor, thereby improving specificity and targeting accuracy [216]. c. The design of intelligent controlled release systems: controlled-release mechanisms, such as redox-driven, enzyme-driven, light-driven, or heat-driven mechanisms, can be employed to enable stimulus-responsive ODN releases under specific circumstances, thereby reducing off-target effects [217]. d. Integration with immunotherapy or chemotherapy: Combining aptamer–ODN chimeras with immunotherapy or chemotherapy could produce synergistic therapeutic effects, ultimately enhancing overall efficacy. Aptamer–ODN chimeras can also be combined with emerging technologies such as CRISPR gene editing technology or nanomedicine to further improve the targeting, controllability, and the efficacy of disease treatment. (2) The optimization of SELEX technologies: With the widespread application of aptamers as targeting ligands, traditional WC-SELEX has been unable to meet actual application needs due to its inherent limitations such as long experimental cycle and complex operation process. To overcome these technical bottlenecks, it is necessary to develop new and efficient aptamer screening systems. For instance, Fluorescence-activated cell sorting-assisted WC-SELEX (FACS-WC-SELEX) combines FACS with WC-SELEX. In this method, the random library is labeled with fluorescein. After incubation and washing, cells are analyzed using a flow cytometer. Upon laser excitation, aptamer-bound cells emit fluorescent signals, which are quantified by the FACS system to isolate positive cells. This method significantly shortens screening time, improves aptamer enrichment, and enhances specificity. Microfluidic chip system-assisted WC-SELEX (MC-WC-SELEX) integrates micro-fluidic technology with WC-SELEX to automate the on-chip SELEX process. Compared to conventional WC-SELEX, MC-WC-SELEX is faster, highly automated, and can complete 15 rounds of selection in just 3 days, while requiring fewer cells and reagents. The 3D culture-assisted WC-SELEX (3D-WC-SELEX) employs target cells cultured in a 3D environment that mimics physiological conditions, thereby enhancing the relevance and selectivity of aptamer screening. Nanomaterial-assisted WC-SELEX (NA-WC-SELEX) incorporates suitable nanomaterials into the selection process to shorten the screening time and improve aptamer performance [30]. Continuous innovation in technologies such as high-throughput screening, AI algorithms, and single-round SELEX technologies is essential to identify more suitable aptamers that meet application needs [31,218,219,220]. (3) The optimization of large-scale production: Long-chain chimeras (>80 nucleotides) are prone to generating truncated products, posing significant challenges for large-scale manufacturing. The production of high-quality, cGMP-grade chimeras remains a major hurdle in therapeutic development. Solving these problems may be done from multiple aspects, such as optimizing the synthesis process, optimizing the purification method, and exploring the enzymatic in vitro synthesis platform [214]. (4) An in-depth study of the intracellular fate of chimeras: For almost all aptamer-ODN chimeras delivery, their fate within the cell, especially the transitions required to cross lipid biolayers, such as to escape from endosomal vesicles, remains elusive [221]. Moreover, there is a lack of quantifications of escape efficiency. Therefore, it is necessary to gain a deeper understanding of the intracellular fate of the chimeras, further study their internalization mechanism, and establish a standardized evaluation system to quantify the differences in endosomal escape rates of different designs.

In summary, we have reviewed recent progress in aptamer–ODN chimeras. As a novel targeted delivery system, aptamer–ODN chimeras offer notable advantages such as precise targeting, a strong cellular internalization ability, and cost-effectiveness, showing broad application potential across multiple therapeutic fields. However, several challenges remain to be addressed in order to promote the development of aptamer–ODN chimeras and expand their applications in biomedicine and related areas, as discussed above. With ongoing advancements in nucleic acid chemistry, delivery system engineering, SELEX technologies, and other relevant fields, the clinical potential of aptamer–ODN chimeras is expected to be fully realized.

## Figures and Tables

**Table 1:** Overview of the research progress of aptamer-siRNA chimera therapy.

ODNs	Aptamer’s Name/Target	Target	Disease	Year	Ref.
siRNA	A9/PMSA	laminA/C	prostate cancer	2006	[51]
	A10/PMSA	PLK1 and BCL2	prostate cancer	2006	[36]
	A10-3.2/PSMA	PLK1	prostate cancer	2009	[52]
	PSMA aptamers/PSMA	Smg-1/Upf-2	colon cancer	2010	[53]
	PSMA aptamers/PSMA	Smg-1/Upf-2	prostate cancer	2013	[54]
	A10-3/PSMA	DNA-PK	prostate cancer	2015	[55]
	PSMA aptamers/PSMA	EGFR and survivin	prostate cancer	2016	[56]
	A10-3.2/PSMA	MDM2	prostate cancer	2022	[37]
	A10-3.2/PSMA	CAT-1	prostate cancer	2022	[57]
	4-1BB aptamer/4-1BB	mTORC1	antitumor immunity	2014	[58]
	4-1BB aptamer/4-1BB	CD25	antitumor immunity	2017	[59]
	gp120 aptamer/gp120 glycoprotein	HIV-1 gag p17	HIV	2008	[60]
	gp120 aptamer/gp120 glycoprotein	HIV-1 tat/rev	HIV	2009	[61]
	gp120 aptamer/gp120 glycoprotein	HIV-1 tat/rev	HIV	2011	[62]
	gp120 aptamer/gp120 glycoprotein	HIV-1 tat/rev	HIV	2011	[63]
	gp120 aptamer/gp120 glycoprotein	HIV-1 tat/rev	HIV	2011	[64]
	gp120 aptamer/gp120 glycoprotein	HIV-1 tat/rev	HIV	2013	[65]
	gp120 aptamer/gp120 glycoprotein	HIV-1 LTR	HIV	2018	[66]
	CCR5 aptamer/CCR5	TNPO3	HIV	2015	[67]
	CD4 aptamer/CD4	HIV genes and/or CCR5	HIV	2011	[68]
	CD4 aptamer/CD4	STAT5b	asthma	2012	[69]
	CD4 aptamer/CD4	HIV-PR	HIV	2012	[70]
	CD4 aptamer/CD4	CCR5	HIV	2013	[71]
	CD8AP17s/CD8	GNLY	immune diseases	2013	[72]
	BAFF-R aptamer/BAFF-R	STAT3	non-Hodgkin’s lymphoma	2013	[73]
	aptamer 32/U87-EGFRvIII	c-Met	gioma	2014	[74]
	aptNCL/nucleolin	SLUG and NRP1	lung cancer	2014	[75]
	AS1411/nucleolin	Bcl-2	breast cancer	2018	[76]
	AS1411/nucleolin	OPN	gioma	2022	[42]
	AS1411/nucleolin	SMG1	antitumor immunity	2022	[77]
	EpCAM aptamer/EpCAM	EpCAM	breast cancer/retinoblastoma	2015	[78]
	EpCAM aptamer/EpCAM	survivin	breast cancer	2015	[79]
	EpCAM aptamer/EpCAM	EpCAM	epithelial cancer	2015	[80]
	EpCAM aptamer/EpCAM	PLK1	breast cancer	2015	[81]
	EpCAM aptamer/EpCAM	D5D	colon cancer	2019	[82]
	EpCAM aptamer/EpCAM	PKCι	breast cancer	2020	[40]
	EpCAM aptamer/EpCAM	D5D	lung cancer	2020	[83]
	HER2 aptamer/HER2	Bcl-2	breast cancer	2012	[84]
	HER2, HER3 aptamer/HER2, HER3	EGFR	breast cancer	2018	[85]
	HER2 aptamer/HER2	EGFR	breast cancer	2018	[86]
	HER2 aptamer/HER2	XBP1	breast cancer	2020	[41]
	EGFR aptamer/EGFR	Bcl-2 and PKC-ι	breast cancer	2019	[87]
	EGFR aptamer/EGFR	XBP1	breast cancer	2021	[39]
	EGFR aptamer/EGFR	KRAS	non-small cell lung cancer	2023	[88]
	ErbB3 aptamer/ErbB3	survivin	breast cancer	2020	[89]
	sgc8c and sgc4f/PTK7	VEGF	leukemia	2016	[90]
	mucin-1 aptamers/mucin-1 (MUC1)	BCL2	multidrug-resistant breast cancer	2017	[91]
	VEGF aptamer/VEGF	notch 3	ovarian cancer	2017	[92]
	GBN/RGNNV coat protein (CP) protein	viral CP	viral nervous necrosis	2020	[93]
	LYGV1c/coat protein (CP) protein	SGIV MCP and VP19	grouper iridovirus infection	2021	[94]
	CTLA4 aptamer/CTLA4	STAT3	colorectal cancer	2014	[95]
	αvβ3 aptamer/αvβ3	EEF2	prostate cancer/cervical cancer/glioblastoma	2013	[96]
	Gint4.T/PDGFRβ	STAT3	glioblastoma	2018	[97]
	Gint4.T/PDGFRβ	STAT3	glioblastoma	2020	[98]
	Gint4.T/PDGFRβ	STAT3	non-small cell lung cancer	2023	[99]
	FB4/TfR	ICAM-1	neuroinflammatory disease	2014	[100]
	IGFIIR aptamer/IGFIIR	PCBP2	antifibrotic	2017	[101]
	monocytes/macrophages aptamer/monocytes/macrophages	ADK	immune diseases	2018	[102]
shRNA	CD40 aptamer/CD40	SMG1	B lymphoma and bone-marrow aplasia	2015	[103]
	CD40 aptamer/CD40	RORγt	immune diseases	2014	[104]
	CD30 aptamer/CD30	RORγt	autoimmune inflammatory diseases	2019	[105]
	S3R3/integrase	HIV-1 tat/rev	HIV	2018	[106]
	A10/PSMA	DNAPK	prostate cancer	2011	[107]

**Table 2:** Overview of the research progress of aptamer-ASO chimera therapy.

ODNs	Aptamer’s Name/Target	Target	Disease	Year	Ref.
ASO	AS1411/nucleolin	luciferase	pancreatic cancer	2012	[132]
	TfR aptamer/TfR	caspase-3	ischemic stroke	2013	[133]
	S6 aptamer/A549	MMP-9	lung cancer	2015	[134]
	AS1411/nucleolin	galectin-1	breast cancer	2016	[135]
	Apt-2/LAMP1	MALAT1	lung cancer	2021	[28]
	AS1411/nucleolin	c-myc	lung cancer	2021	[136]
	PA9-1/PD-L1	PD-L1	antitumor immunity	2023	[137]
	SApt/SARS-CoV-2 spike protein	SARS-CoV-2 nucleocapsid	SARS-CoV-2	2023	[138]

**Table 3:** Overview of the research progress of aptamer-miRNA chimera therapy.

ODNs	Aptamer’s Name/Target	Target	Disease	Year	Ref.
miRNA	CD133 aptamer/CD133	Anti-miR21	triple-negative breast cancer	2019	[117]
	EGFR aptamer/EGFR	Anti-miR21	breast cancer	2015	[116]
	EGFR aptamer/EGFR	Anti-miR21	breast cancer	2021	[154]
	A549 aptamer/A549	miR-301b-3p	lung adenocarcinoma	2023	[118]
	α9-nAChR aptamer/α9-nAChR	Anti-miR21	breast cancer	2023	[115]
	MUC1 aptamer/MUC1	PTEN	ovarian cancer	2013	[155]
	GL21.T/Axl	HMGA2	non-small cell lung cancer	2014	[153]
	GL21.T/Axl	miR-222	glioblastoma	2015	[147]
	GL21.T and Gint4.T/PDGFRβ and Axl	miR-137 and anti-miR-10b	glioblastoma	2016	[151]
	GL21.T/Axl	PED/PEA-15	non-small cell lung cancer	2016	[152]
	GL21.T/Axl	AXL	non-small cell lung cancer	2018	[150]
	GL21.T/Axl	Axl and the miR-137	non-small cell lung cancer	2019	[156]
	AS1411/nucleolin	c-raf-1	non-small cell lung cancer	2018	[157]
	AS1411/nucleolin	miRNA let-7d	gastric cancer	2019	[149]
	A10–3.2/PSMA	Bcl-2, cyclin D1, Wnt3a	prostate cancer	2011	[158]
	cKIT aptamer/cKIT	Ezh2 and Bak1	breast cancer	2023	[159]
	apt69.T/B cell maturation antigen	miR-137 and miR-122	multiple myeloma	2019	[146]

**Table 4:** Overview of the research progress of aptamer-saRNA/decoy chimera therapy.

ODNs	Aptamer’s Name/Target	Target	Disease	Year	Ref.
saRNA	P19/P1 aptamer/PDAC	*C/EBPα*	pancreatic cancer	2016	[167]
	TR14/hTfR	*C/EBP*α	prostate cancer	2019	[168]
	A10-3.2/PSMA	*DPYSL3*	prostate cancer	2016	[169]
	m12-3773/clusterin and 1-717/TMED6	*XIAP*	prostate cancer	2022	[170]
decoy	c2.min/hTfR	NF-κB	pancreatic cancer	2015	[171]
	GS-24/TfR	NF-κB	cerebrovascular inflammation	2015	[172]

**Table 5:** Overview of the research progress of aptamer-CpG/sgRNA chimera therapy.

ODNs	Aptamer’s Name/Target	Target	Disease	Year	Ref.
cpg	IL-4Rα/IL-4Rα receptor	CpG-induced pathways	antitumor immunity	2017	[196]
	AS1411/nucleolin	CpG-induced pathways	antitumor immunity	2018	[181]
	AS1411/nucleolin	CpG-induced pathways	antitumor immunity	2024	[197]
	Aapt/ATP	CpG-induced pathways	antitumor immunity	2021	[198]
	ROX40 aptamer/ROX40	CpG-induced pathways	antitumor immunity	2025	[199]
	MUC1 aptamer/MUC1	CpG-induced pathways	antitumor immunity	2019	[200]
	PD-L1 aptamer/PD-L1	CpG-induced pathways	metastatic lung cancer	2023	[201]
	PD-L1 aptamer/PD-L1	CpG-induced pathways	antitumor immunity	2022	[202]
	PD-L1 aptamer/PD-L1	CpG-induced pathways	post-operative tumor recurrence	2023	[182]
others	com/Com	sgRNA	duchenne muscular dystrophy	2021	[179]
	PD-1 aptamer/PD-1	sgRNA	antitumor immunity	2019	[190]
	SS30/CD123	sgRNA	acute myeloid leukemia	2019	[180]
	NCL-APT/nucleolin	survivin	retinoblastoma	2021	[203]
	MUC1 aptamer/MUC1	Egr-1	breast cancer	2020	[204]
	Sgc8/PTK7	MET	lung cancer	2020	[205]
	CD44-EpCAM aptamer/CD44 and EpCAM	CD44 and EpCAM	ovarian cancer	2017	[206]
	TfR-aptamer/TfR	Tau	tauopathy	2020	[207]

## Data Availability

No new data were created or analyzed in this study.

## Abbreviations

The following abbreviations are used in this manuscript:

ODN	oligonucleotide
siRNA	small interfering RNA
saRNA	small activating RNA
miRNA	microRNA
sgRNA	single guide RNA
shRNA	short hairpin RNA
ASO	antisense oligonucleotide
ApDCs	aptamer-drug conjugates
AMO	Anti-miRNA ODN
RNAi	RNA interference
Ago 2	Argonaute 2
RNase H	Ribonuclease H
RIBOTAC	RNA-inducible ribonuclease-targeted chimera
PROTAC	protein degradation-targeted chimera
3′-UTR	3′ Untranslated Region
FDA	US Food and Drug Administration
EMA	European Medicines Agency
SELEX	systematic evolution of ligands by exponential enrichment
WC-SELEX	whole cell SELEX
FACS-WC-SELEX	fluorescence-activated cell sorting-assisted WC-SELEX
MC-WC-SELEX	microfluidic chip system-assisted WC-SELEX
3D-WC-SELEX	three-dimensional culture-assisted WC-SELEX
NA-WC-SELEX	nanomaterial-assisted WC-SELEX
RME	receptor-mediated endocytosis
CME	clathrin-mediated endocytosis
CvME	caveolae-mediated endocytosis
DSB	double-stranded DNA break
NHEJ	non-homologous end-joining
HDR	homology-directed repair
3WJ	the three-way junction motif
pRNA	packaging RNA
VEGF	vascular endothelial growth factor
TfR	transferrin receptor
PTK7	protein tyrosine kinase 7
mCRPC	metastatic castration-resistant prostate cancer
PSMA	prostate-specific membrane antigen
Bcl-2	B-cell lymphoma 2
Plk1	polo-like kinase 1
DOX	doxorubicin
SARS-CoV-2	severe acute respiratory syndrome coronavirus 2
CEBPA	CCAAT/Enhancer Binding Protein Alpha
PDAC	pancreatic ductal adenocarcinoma
NF-κB	nuclear factor κB
EGFR	epidermal growth factor receptor
PD-L1	programmed death-ligand 1
PD-1	programmed cell death protein 1
RCA	rolled-loop amplification
VSV-G	vesicular stomatitis virus G glycoprotein
ABP	aptamer-binding protein
AML	acute myeloid leukemia
TLR9	Toll-like receptor 9
DSPE	1,2-distearoyl-sn-glycero-3-phospho-ethanolamine
HDL	high-density lipoprotein
Apo-AI	Apolipoprotein AI
SRBI	scavenger receptor class B type I
NIR	near-infrared

## References

[1] Roberts T.C., Langer R., Wood M.J.A. (2020). Advances in oligonucleotide drug delivery. Nat. Rev. Drug Discov..

[2] Mangla P., Vicentini Q., Biscans A. (2023). Therapeutic Oligonucleotides: An Outlook on Chemical Strategies to Improve Endosomal Trafficking. Cells.

[3] Vinjamuri B.P., Pan J., Peng P. (2024). A Review on Commercial Oligonucleotide Drug Products. J. Pharm. Sci..

[4] Anand P., Zhang Y., Patil S., Kaur K. (2025). Metabolic Stability and Targeted Delivery of Oligonucleotides: Advancing RNA Therapeutics Beyond The Liver. J. Med. Chem..

[5] Fabrega C., Avino A., Navarro N., Jorge A.F., Grijalvo S., Eritja R. (2023). Lipid and Peptide-Oligonucleotide Conjugates for Therapeutic Purposes: From Simple Hybrids to Complex Multifunctional Assemblies. Pharmaceutics.

[6] Khairnar P., Kolipaka T., Pandey G., Phatale V., Shah S., Srinivasarao D.A., Saraf S., Srivastava S. (2024). Nanosponge-Mediated Oligonucleotide Delivery: A Cutting-Edge Technology Towards Cancer Management. J. Drug Deliv. Sci. Technol..

[7] Nuzzo S., Roscigno G., Affinito A., Ingenito F., Quintavalle C., Condorelli G. (2019). Potential and Challenges of Aptamers as Specific Carriers of Therapeutic Oligonucleotides for Precision Medicine in Cancer. Cancers.

[8] Sun H., Zhu X., Lu P.Y., Rosato R.R., Tan W., Zu Y. (2014). Oligonucleotide aptamers: New tools for targeted cancer therapy. Mol. Ther. Nucleic Acids.

[9] Fan R., Tao X., Zhai X., Zhu Y., Li Y., Chen Y., Dong D., Yang S., Lv L. (2023). Application of aptamer-drug delivery system in the therapy of breast cancer. Biomed. Pharmacother..

[10] Soldevilla M.M., Meraviglia-Crivelli de Caso D., Menon A.P., Pastor F. (2018). Aptamer-iRNAs as Therapeutics for Cancer Treatment. Pharmaceuticals.

[11] Zhou J., Rossi J. (2017). Aptamers as targeted therapeutics: Current potential and challenges. Nat. Rev. Drug Discov..

[12] Moradi Z., Abnous K., Taghdisi S.M., Zamanian J., Moshiri M., Etemad D., Etemad L., Kesharwani P., Sahebkar A. (2025). Designing multivalent aptamers: Recent advancements in diagnostic and therapeutic approaches for cancer treatment. J. Drug Deliv. Sci. Technol..

[13] Elskens J.P., Elskens J.M., Madder A. (2020). Chemical Modification of Aptamers for Increased Binding Affinity in Diagnostic Applications: Current Status and Future Prospects. Int. J. Mol. Sci..

[14] Meng H.M., Liu H., Kuai H., Peng R., Mo L., Zhang X.B. (2016). Aptamer-integrated DNA nanostructures for biosensing, bioimaging and cancer therapy. Chem. Soc. Rev..

[15] U A.P., Raj G., John J., Mohan K.M., John F., George J. (2023). Aptamers: Features, Synthesis and Applications. Chem. Biodivers..

[16] Zhang Y., Lai B.S., Juhas M. (2019). Recent Advances in Aptamer Discovery and Applications. Molecules.

[17] Zheng X., Huang Z., Zhang Q., Li G., Song M., Peng R. (2025). Aptamer-functionalized nucleic acid nanotechnology for biosensing, bioimaging and cancer therapy. Nanoscale.

[18] Bege M., Ghanem Kattoub R., Borbas A. (2025). The 20th Anniversary of Pegaptanib (MacugenTM), the First Approved Aptamer Medicine: History, Recent Advances and Future Prospects of Aptamers in Therapy. Pharmaceutics.

[19] He S., Du Y., Tao H., Duan H. (2023). Advances in aptamer-mediated targeted delivery system for cancer treatment. Int. J. Biol. Macromol..

[20] Di Y., Wang P., Li C., Xu S., Tian Q., Wu T., Tian Y., Gao L. (2020). Design, Bioanalytical, and Biomedical Applications of Aptamer-Based Hydrogels. Front. Med..

[21] Moosavian S.A., Sahebkar A. (2019). Aptamer-functionalized liposomes for targeted cancer therapy. Cancer Lett..

[22] Sheikh A., Md S., Alhakamy N.A., Kesharwani P. (2022). Recent development of aptamer conjugated chitosan nanoparticles as cancer therapeutics. Int. J. Pharm..

[23] Urmi R., Banerjee P., Singh M., Singh R., Chhillar S., Sharma N., Chandra A., Singh N., Qamar I. (2024). Revolutionizing biomedicine: Aptamer-based nanomaterials and nanodevices for therapeutic applications. Biotechnol. Rep..

[24] Yan J., Gao T., Lu Z., Yin J., Zhang Y., Pei R. (2021). Aptamer-Targeted Photodynamic Platforms for Tumor Therapy. ACS Appl. Mater. Interfaces.

[25] Alamudi S.H., Kimoto M., Hirao I. (2021). Uptake mechanisms of cell-internalizing nucleic acid aptamers for applications as pharmacological agents. RSC Med. Chem..

[26] Wan L.Y., Yuan W.F., Ai W.B., Ai Y.W., Wang J.J., Chu L.Y., Zhang Y.Q., Wu J.F. (2019). An exploration of aptamer internalization mechanisms and their applications in drug delivery. Expert. Opin. Drug Deliv..

[27] Lv C., Yang C., Ding D., Sun Y., Wang R., Han D., Tan W. (2019). Endocytic Pathways and Intracellular Transport of Aptamer-Drug Conjugates in Live Cells Monitored by Single-Particle Tracking. Anal. Chem..

[28] Tanaka K., Okuda T., Kasahara Y., Obika S. (2021). Base-modified aptamers obtained by cell-internalization SELEX facilitate cellular uptake of an antisense oligonucleotide. Mol. Ther. Nucleic Acids.

[29] Gopinath S.C., Lakshmipriya T., Chen Y., Arshad M.K., Kerishnan J.P., Ruslinda A.R., Al-Douri Y., Voon C.H., Hashim U. (2016). Cell-targeting aptamers act as intracellular delivery vehicles. Appl. Microbiol. Biotechnol..

[30] Duan Y., Zhang C., Wang Y., Chen G. (2022). Research progress of whole-cell-SELEX selection and the application of cell-targeting aptamer. Mol. Biol. Rep..

[31] Liu S., Li X., Gao H., Chen J., Jiang H. (2025). Progress in Aptamer Research and Future Applications. ChemistryOpen.

[32] Ni S., Zhuo Z., Pan Y., Yu Y., Li F., Liu J., Wang L., Wu X., Li D., Wan Y. (2021). Recent Progress in Aptamer Discoveries and Modifications for Therapeutic Applications. ACS Appl. Mater. Interfaces.

[33] Wu L., Wang Y., Xu X., Liu Y., Lin B., Zhang M., Zhang J., Wan S., Yang C., Tan W. (2021). Aptamer-Based Detection of Circulating Targets for Precision Medicine. Chem. Rev..

[34] Ali M.H., Elsherbiny M.E., Emara M. (2019). Updates on Aptamer Research. Int. J. Mol. Sci..

[35] Pandey S.K., Parul M., Santhanam M. (2024). Aptamer-guided Selective Delivery of Therapeutics to Breast Cancer Cells Expressing Specific Biomarkers. Curr. Cancer Ther. Rev..

[36] McNamara J.O., Andrechek E.R., Wang Y., Viles K.D., Rempel R.E., Gilboa E., Sullenger B.A., Giangrande P.H. (2006). Cell type-specific delivery of siRNAs with aptamer-siRNA chimeras. Nat. Biotechnol..

[37] Jiao Y., Xu P., Luan S., Wang X., Gao Y., Zhao C., Fu P. (2022). Molecular imaging and treatment of PSMA-positive prostate cancer with (99m)Tc radiolabeled aptamer-siRNA chimeras. Nucl. Med. Biol..

[38] Rosch J.C., Hoogenboezem E.N., Sorets A.G., Duvall C.L., Lippmann E.S. (2022). Albumin-Binding Aptamer Chimeras for Improved siRNA Bioavailability. Cell. Mol. Bioeng..

[39] Zhang L., Mu C., Zhang T., Yang D., Wang C., Chen Q., Tang L., Fan L., Liu C., Shen J. (2021). Development of targeted therapy therapeutics to sensitize triple-negative breast cancer chemosensitivity utilizing bacteriophage phi29 derived packaging RNA. J. Nanobiotechnol..

[40] Rehmani H., Li Y., Li T., Padia R., Calbay O., Jin L., Chen H., Huang S. (2020). Addiction to protein kinase Ci due to PRKCI gene amplification can be exploited for an aptamer-based targeted therapy in ovarian cancer. Signal Transduct. Target. Ther..

[41] Zhang L., Mu C., Zhang T., Wang Y., Wang Y., Fan L., Liu C., Chen H., Shen J., Wei K. (2020). Systemic Delivery of Aptamer-Conjugated XBP1 siRNA Nanoparticles for Efficient Suppression of HER2+ Breast Cancer. ACS Appl. Mater. Interfaces.

[42] Wei J., Song R., Sabbagh A., Marisetty A., Shukla N., Fang D., Najem H., Ott M., Long J., Zhai L. (2022). Cell-directed aptamer therapeutic targeting for cancers including those within the central nervous system. Oncoimmunology.

[43] Velema W.A., Lu Z. (2023). Chemical RNA Cross-Linking: Mechanisms, Computational Analysis, and Biological Applications. JACS Au.

[44] Mirzaei S., Paskeh M.D.A., Entezari M., Bidooki S.H., Ghaleh V.J., Rezaei S., Hejazi E.S., Kakavand A., Behroozaghdam M., Movafagh A. (2023). siRNA and targeted delivery systems in breast cancer therapy. Clin. Transl. Oncol..

[45] Saw P.E., Song E.W. (2020). siRNA therapeutics: A clinical reality. Sci. China Life Sci..

[46] Alshaer W., Zureigat H., Al Karaki A., Al-Kadash A., Gharaibeh L., Hatmal M.M., Aljabali A.A.A., Awidi A. (2021). siRNA: Mechanism of action, challenges, and therapeutic approaches. Eur. J. Pharmacol..

[47] Friedrich M., Aigner A. (2022). Therapeutic siRNA: State-of-the-Art and Future Perspectives. BioDrugs.

[48] Zhang M.M., Bahal R., Rasmussen T.P., Manautou J.E., Zhong X.B. (2021). The growth of siRNA-based therapeutics: Updated clinical studies. Biochem. Pharmacol..

[49] Dong Y., Siegwart D.J., Anderson D.G. (2019). Strategies, design, and chemistry in siRNA delivery systems. Adv. Drug Deliv. Rev..

[50] Ku S.H., Jo S.D., Lee Y.K., Kim K., Kim S.H. (2016). Chemical and structural modifications of RNAi therapeutics. Adv. Drug Deliv. Rev..

[51] Chu T.C., Twu K.Y., Ellington A.D., Levy M. (2006). Aptamer mediated siRNA delivery. Nucleic Acids Res..

[52] Dassie J.P., Liu X.Y., Thomas G.S., Whitaker R.M., Thiel K.W., Stockdale K.R., Meyerholz D.K., McCaffrey A.P., McNamara J.O., Giangrande P.H. (2009). Systemic administration of optimized aptamer-siRNA chimeras promotes regression of PSMA-expressing tumors. Nat. Biotechnol..

[53] Pastor F., Kolonias D., Giangrande P.H., Gilboa E. (2010). Induction of tumour immunity by targeted inhibition of nonsense-mediated mRNA decay. Nature.

[54] Gilboa E. (2013). Expression of new antigens on tumor cells by inhibiting nonsense-mediated mRNA decay. Immunol. Res..

[55] Ni X., Zhang Y., Zennami K., Castanares M., Mukherjee A., Raval R.R., Zhou H., DeWeese T.L., Lupold S.E. (2015). Systemic Administration and Targeted Radiosensitization via Chemically Synthetic Aptamer-siRNA Chimeras in Human Tumor Xenografts. Mol. Cancer Ther..

[56] Liu H.Y., Yu X., Liu H., Wu D., She J.X. (2016). Co-targeting EGFR and survivin with a bivalent aptamer-dual siRNA chimera effectively suppresses prostate cancer. Sci. Rep..

[57] Guo L., Shi D., Shang M., Sun X., Meng D., Liu X., Zhou X., Li J. (2022). Utilizing RNA nanotechnology to construct negatively charged and ultrasound-responsive nanodroplets for targeted delivery of siRNA. Drug Deliv..

[58] Berezhnoy A., Castro I., Levay A., Malek T.R., Gilboa E. (2014). Aptamer-targeted inhibition of mTOR in T cells enhances antitumor immunity. J. Clin. Investig..

[59] Rajagopalan A., Berezhnoy A., Schrand B., Puplampu-Dove Y., Gilboa E. (2017). Aptamer-Targeted Attenuation of IL-2 Signaling in CD8(+) T Cells Enhances Antitumor Immunity. Mol. Ther..

[60] Zhou J., Li H., Li S., Zaia J., Rossi J.J. (2008). Novel dual inhibitory function aptamer-siRNA delivery system for HIV-1 therapy. Mol. Ther..

[61] Zhou J., Swiderski P., Li H., Zhang J., Neff C.P., Akkina R., Rossi J.J. (2009). Selection, characterization and application of new RNA HIV gp 120 aptamers for facile delivery of Dicer substrate siRNAs into HIV infected cells. Nucleic Acids Res..

[62] Zhou J., Li H., Zhang J., Piotr S., Rossi J. (2011). Development of cell-type specific anti-HIV gp120 aptamers for siRNA delivery. J. Vis. Exp. JoVE.

[63] Zhou J., Shu Y., Guo P., Smith D.D., Rossi J.J. (2011). Dual functional RNA nanoparticles containing phi29 motor pRNA and anti-gp120 aptamer for cell-type specific delivery and HIV-1 inhibition. Methods.

[64] Neff C.P., Zhou J., Remling L., Kuruvilla J., Zhang J., Li H., Smith D.D., Swiderski P., Rossi J.J., Akkina R. (2011). An aptamer-siRNA chimera suppresses HIV-1 viral loads and protects from helper CD4(+) T cell decline in humanized mice. Sci. Transl. Med..

[65] Zhou J., Neff C.P., Swiderski P., Li H., Smith D.D., Aboellail T., Remling-Mulder L., Akkina R., Rossi J.J. (2013). Functional in vivo delivery of multiplexed anti-HIV-1 siRNAs via a chemically synthesized aptamer with a sticky bridge. Mol. Ther..

[66] Zhou J., Lazar D., Li H., Xia X., Satheesan S., Charlins P., O’Mealy D., Akkina R., Saayman S., Weinberg M.S. (2018). Receptor-targeted aptamer-siRNA conjugate-directed transcriptional regulation of HIV-1. Theranostics.

[67] Zhou J., Satheesan S., Li H., Weinberg M.S., Morris K.V., Burnett J.C., Rossi J.J. (2015). Cell-specific RNA aptamer against human CCR5 specifically targets HIV-1 susceptible cells and inhibits HIV-1 infectivity. Chem. Biol..

[68] Wheeler L.A., Trifonova R., Vrbanac V., Basar E., McKernan S., Xu Z., Seung E., Deruaz M., Dudek T., Einarsson J.I. (2011). Inhibition of HIV transmission in human cervicovaginal explants and humanized mice using CD4 aptamer-siRNA chimeras. J. Clin. Investig..

[69] Qiu C., Peng W.K., Shi F., Zhang T. (2012). Bottom-up assembly of RNA nanoparticles containing phi29 motor pRNA to silence the asthma STAT5b gene. Genet. Mol. Res..

[70] Zhu Q., Shibata T., Kabashima T., Kai M. (2012). Inhibition of HIV-1 protease expression in T cells owing to DNA aptamer-mediated specific delivery of siRNA. Eur. J. Med. Chem..

[71] Wheeler L.A., Vrbanac V., Trifonova R., Brehm M.A., Gilboa-Geffen A., Tanno S., Greiner D.L., Luster A.D., Tager A.M., Lieberman J. (2013). Durable knockdown and protection from HIV transmission in humanized mice treated with gel-formulated CD4 aptamer-siRNA chimeras. Mol. Ther..

[72] Wang C.W., Chung W.H., Cheng Y.F., Ying N.W., Peck K., Chen Y.T., Hung S.I. (2013). A new nucleic acid-based agent inhibits cytotoxic T lymphocyte-mediated immune disorders. J. Allergy Clin. Immunol..

[73] Zhou J., Tiemann K., Chomchan P., Alluin J., Swiderski P., Burnett J., Zhang X., Forman S., Chen R., Rossi J. (2013). Dual functional BAFF receptor aptamers inhibit ligand-induced proliferation and deliver siRNAs to NHL cells. Nucleic Acids Res..

[74] Zhang X., Liang H., Tan Y., Wu X., Li S., Shi Y. (2014). A U87-EGFRvIII cell-specific aptamer mediates small interfering RNA delivery. Biomed. Rep..

[75] Lai W.Y., Wang W.Y., Chang Y.C., Chang C.J., Yang P.C., Peck K. (2014). Synergistic inhibition of lung cancer cell invasion, tumor growth and angiogenesis using aptamer-siRNA chimeras. Biomaterials.

[76] Cheng H., Hong S., Wang Z., Sun N., Wang T., Zhang Y., Chen H., Pei R. (2018). Self-assembled RNAi nanoflowers via rolling circle transcription for aptamer-targeted siRNA delivery. J. Mater. Chem. B.

[77] Meraviglia-Crivelli D., Villanueva H., Menon A.P., Zheleva A., Moreno B., Villalba-Esparza M., Pastor F. (2022). A pan-tumor-siRNA aptamer chimera to block nonsense-mediated mRNA decay inflames and suppresses tumor progression. Mol. Ther. Nucleic Acids.

[78] Subramanian N., Kanwar J.R., Athalya P.K., Janakiraman N., Khetan V., Kanwar R.K., Eluchuri S., Krishnakumar S. (2015). EpCAM aptamer mediated cancer cell specific delivery of EpCAM siRNA using polymeric nanocomplex. J. Biomed. Sci..

[79] Wang T., Gantier M.P., Xiang D., Bean A.G., Bruce M., Zhou S.F., Khasraw M., Ward A., Wang L., Wei M.Q. (2015). EpCAM Aptamer-mediated Survivin Silencing Sensitized Cancer Stem Cells to Doxorubicin in a Breast Cancer Model. Theranostics.

[80] Subramanian N., Kanwar J.R., Kanwar R.K., Sreemanthula J., Biswas J., Khetan V., Krishnakumar S. (2015). EpCAM Aptamer-siRNA Chimera Targets and Regress Epithelial Cancer. PLoS ONE.

[81] Gilboa-Geffen A., Hamar P., Le M.T., Wheeler L.A., Trifonova R., Petrocca F., Wittrup A., Lieberman J. (2015). Gene Knockdown by EpCAM Aptamer-siRNA Chimeras Suppresses Epithelial Breast Cancers and Their Tumor-Initiating Cells. Mol. Cancer Ther..

[82] Xu Y., Pang L., Wang H., Xu C., Shah H., Guo P., Shu D., Qian S.Y. (2019). Specific delivery of delta-5-desaturase siRNA via RNA nanoparticles supplemented with dihomo-gamma-linolenic acid for colon cancer suppression. Redox Biol..

[83] Pang L., Shah H., Wang H., Shu D., Qian S.Y., Sathish V. (2020). EpCAM-Targeted 3WJ RNA Nanoparticle Harboring Delta-5-Desaturase siRNA Inhibited Lung Tumor Formation via DGLA Peroxidation. Mol. Ther. Nucleic Acids.

[84] Thiel K.W., Hernandez L.I., Dassie J.P., Thiel W.H., Liu X., Stockdale K.R., Rothman A.M., Hernandez F.J., McNamara J.O., Giangrande P.H. (2012). Delivery of chemo-sensitizing siRNAs to HER2+-breast cancer cells using RNA aptamers. Nucleic Acids Res..

[85] Yu X., Ghamande S., Liu H., Xue L., Zhao S., Tan W., Zhao L., Tang S.C., Wu D., Korkaya H. (2018). Targeting EGFR/HER2/HER3 with a Three-in-One Aptamer-siRNA Chimera Confers Superior Activity against HER2(+) Breast Cancer. Mol. Ther. Nucleic Acids.

[86] Xue L., Maihle N.J., Yu X., Tang S.C., Liu H.Y. (2018). Synergistic Targeting HER2 and EGFR with Bivalent Aptamer-siRNA Chimera Efficiently Inhibits HER2-Positive Tumor Growth. Mol. Pharm..

[87] Kim M.W., Jeong H.Y., Kang S.J., Jeong I.H., Choi M.J., You Y.M., Im C.S., Song I.H., Lee T.S., Lee J.S. (2019). Anti-EGF Receptor Aptamer-Guided Co-Delivery of Anti-Cancer siRNAs and Quantum Dots for Theranostics of Triple-Negative Breast Cancer. Theranostics.

[88] Yang L., Li Z., Binzel D.W., Guo P., Williams T.M. (2023). Targeting oncogenic KRAS in non-small cell lung cancer with EGFR aptamer-conjugated multifunctional RNA nanoparticles. Mol. Ther. Nucleic Acids.

[89] Xu X., Li L., Li X., Tao D., Zhang P., Gong J. (2020). Aptamer-protamine-siRNA nanoparticles in targeted therapy of ErbB3 positive breast cancer cells. Int. J. Pharm..

[90] Ren K., Liu Y., Wu J., Zhang Y., Zhu J., Yang M., Ju H. (2016). A DNA dual lock-and-key strategy for cell-subtype-specific siRNA delivery. Nat. Commun..

[91] Jeong H., Lee S.H., Hwang Y., Yoo H., Jung H., Kim S.H., Mok H. (2017). Multivalent Aptamer-RNA Conjugates for Simple and Efficient Delivery of Doxorubicin/siRNA into Multidrug-Resistant Cells. Macromol. Biosci..

[92] Chen Y., Xu M., Guo Y., Tu K., Wu W., Wang J., Tong X., Wu W., Qi L., Shi D. (2017). Targeted chimera delivery to ovarian cancer cells by heterogeneous gold magnetic nanoparticle. Nanotechnology.

[93] Zhou L., Wang S., Yu Q., Wei S., Liu M., Wei J., Huang Y., Huang X., Li P., Qin Q. (2020). Characterization of Novel Aptamers Specifically Directed to Red-Spotted Grouper Nervous Necrosis Virus (RGNNV)-Infected Cells for Mediating Targeted siRNA Delivery. Front. Microbiol..

[94] Yu Q., Li W., Liu M., Li M., Zhuo X., Feng L., Wang G., Li P. (2021). Aptamer-mediated targeted siRNA delivery against grouper iridovirus infection. Aquaculture.

[95] Herrmann A., Priceman S.J., Swiderski P., Kujawski M., Xin H., Cherryholmes G.A., Zhang W., Zhang C., Lahtz C., Kowolik C. (2014). CTLA4 aptamer delivers STAT3 siRNA to tumor-associated and malignant T cells. J. Clin. Investig..

[96] Hussain A.F., Tur M.K., Barth S. (2013). An aptamer-siRNA chimera silences the eukaryotic elongation factor 2 gene and induces apoptosis in cancers expressing alphavbeta3 integrin. Nucleic Acid Ther..

[97] Esposito C.L., Nuzzo S., Catuogno S., Romano S., de Nigris F., de Franciscis V. (2018). STAT3 Gene Silencing by Aptamer-siRNA Chimera as Selective Therapeutic for Glioblastoma. Mol. Ther. Nucleic Acids.

[98] Esposito C.L., Nuzzo S., Ibba M.L., Ricci-Vitiani L., Pallini R., Condorelli G., Catuogno S., de Franciscis V. (2020). Combined Targeting of Glioblastoma Stem-Like Cells by Neutralizing RNA-Bio-Drugs for STAT3. Cancers.

[99] Ibba M.L., Ciccone G., Rotoli D., Coppola G., Fiorelli A., Catuogno S., Esposito C.L. (2023). STAT3 silencing by an aptamer-based strategy hampers the crosstalk between NSCLC cells and cancer-associated fibroblasts. Mol. Ther. Nucleic Acids.

[100] Hu J., Xiao F., Hao X., Bai S., Hao J. (2014). Inhibition of monocyte adhesion to brain-derived endothelial cells by dual functional RNA chimeras. Mol. Ther. Nucleic Acids.

[101] Chen Z., Liu H., Jain A., Zhang L., Liu C., Cheng K. (2017). Discovery of Aptamer Ligands for Hepatic Stellate Cells Using SELEX. Theranostics.

[102] Chen Y., Cao J., Cai G., Chen W., Zhou Z. (2018). Aptamer/Adenosine Kinase Chimera Promotes Angiogenesis Through Regulating M2-Type Monocytes/Macrophages Polarization. J. Biomater. Tissue Eng..

[103] Soldevilla M.M., Villanueva H., Bendandi M., Inoges S., Lopez-Diaz de Cerio A., Pastor F. (2015). 2-fluoro-RNA oligonucleotide CD40 targeted aptamers for the control of B lymphoma and bone-marrow aplasia. Biomaterials.

[104] Song P., Chou Y.K., Zhang X., Meza-Romero R., Yomogida K., Benedek G., Chu C.Q. (2014). CD4 aptamer-RORgammat shRNA chimera inhibits IL-17 synthesis by human CD4(+) T cells. Biochem. Biophys. Res. Commun..

[105] Shi X., Song P., Tao S., Zhang X., Chu C.Q. (2019). Silencing RORgammat in Human CD4(+) T cells with CD30 aptamer-RORgammat shRNA Chimera. Sci. Rep..

[106] Pang K.M., Castanotto D., Li H., Scherer L., Rossi J.J. (2018). Incorporation of aptamers in the terminal loop of shRNAs yields an effective and novel combinatorial targeting strategy. Nucleic Acids Res..

[107] Ni X., Zhang Y., Ribas J., Chowdhury W.H., Castanares M., Zhang Z., Laiho M., DeWeese T.L., Lupold S.E. (2011). Prostate-targeted radiosensitization via aptamer-shRNA chimeras in human tumor xenografts. J. Clin. Investig..

[108] Zeng T., Xie Y., Chai K., Sang H. (2024). The Application of Prostate Specific Membrane Antigen in the Diagnosis and Treatment of Prostate Cancer: Status and Challenge. OncoTargets Ther..

[109] Cavalu S., Abdelhamid A.M., Saber S., Elmorsy E.A., Hamad R.S., Abdel-Reheim M.A., Yahya G., Salama M.M. (2024). Cell cycle machinery in oncology: A comprehensive review of therapeutic targets. FASEB J..

[110] Kaloni D., Diepstraten S.T., Strasser A., Kelly G.L. (2023). BCL-2 protein family: Attractive targets for cancer therapy. Apoptosis.

[111] Wullner U., Neef I., Eller A., Kleines M., Tur M.K., Barth S. (2008). Cell-specific induction of apoptosis by rationally designed bivalent aptamer-siRNA transcripts silencing eukaryotic elongation factor 2. Curr. Cancer Drug Targets.

[112] Xue L., Yu X., Zhao L., Garrett A., Wu D., Liu H.Y. (2024). Targeted Delivery of AR-V7 siRNA with Bivalent PSMA Aptamers Effectively Suppresses the Growth of Enzalutamide-Resistant Prostate Cancer. Mol. Pharm..

[113] Ye P., Wang Y., Li R., Chen W., Wan L., Cai P. (2022). The HER family as therapeutic targets in colorectal cancer. Crit. Rev. Oncol. Hematol..

[114] Jacobsen H.J., Poulsen T.T., Dahlman A., Kjaer I., Koefoed K., Sen J.W., Weilguny D., Bjerregaard B., Andersen C.R., Horak I.D. (2015). Pan-HER, an Antibody Mixture Simultaneously Targeting EGFR, HER2, and HER3, Effectively Overcomes Tumor Heterogeneity and Plasticity. Clin. Cancer Res..

[115] Liao Y.C., Cheng T.C., Tu S.H., Chang J., Guo P., Chen L.C., Ho Y.S. (2023). Tumor targeting and therapeutic assessments of RNA nanoparticles carrying alpha9-nAChR aptamer and anti-miR-21 in triple-negative breast cancers. Mol. Ther. Nucleic Acids.

[116] Shu D., Li H., Shu Y., Xiong G., Carson W.E., Haque F., Xu R., Guo P. (2015). Systemic Delivery of Anti-miRNA for Suppression of Triple Negative Breast Cancer Utilizing RNA Nanotechnology. ACS Nano.

[117] Yin H., Xiong G., Guo S., Xu C., Xu R., Guo P., Shu D. (2019). Delivery of Anti-miRNA for Triple-Negative Breast Cancer Therapy Using RNA Nanoparticles Targeting Stem Cell Marker CD133. Mol. Ther..

[118] Zhao J., Niu N., Yang F., Liu H., Qi W. (2023). Preparation, characterisation, and in vitro cancer-suppression function of RNA nanoparticles carrying miR-301b-3p Inhibitor. IET Nanobiotechnol..

[119] Xu C., Haque F., Jasinski D.L., Binzel D.W., Shu D., Guo P. (2018). Favorable biodistribution, specific targeting and conditional endosomal escape of RNA nanoparticles in cancer therapy. Cancer Lett..

[120] Liu X., Duan D., Wang Y., Liu J., Duan D. (2022). Advancements in 3WJ-based RNA nanotechnology and its application for cancer diagnosis and therapy. Front. Biosci..

[121] Isenmann M., Stoddart M.J., Schmelzeisen R., Gross C., Della Bella E., Rothweiler R.M. (2023). Basic Principles of RNA Interference: Nucleic Acid Types and In Vitro Intracellular Delivery Methods. Micromachines.

[122] Tsai H.C., Pietrobon V., Peng M., Wang S., Zhao L., Marincola F.M., Cai Q. (2022). Current strategies employed in the manipulation of gene expression for clinical purposes. J. Transl. Med..

[123] Shalbi F., Ali A.R. (2024). A mini-review on integrase inhibitors: The cornerstone of next-generation HIV treatment. Eur. J. Med. Chem..

[124] Scoles D.R., Minikel E.V., Pulst S.M. (2019). Antisense oligonucleotides: A primer. Neurol. Genet..

[125] Crooke S.T., Baker B.F., Crooke R.M., Liang X.H. (2021). Antisense technology: An overview and prospectus. Nat. Rev. Drug Discov..

[126] Dhuri K., Bechtold C., Quijano E., Pham H., Gupta A., Vikram A., Bahal R. (2020). Antisense Oligonucleotides: An Emerging Area in Drug Discovery and Development. J. Clin. Med..

[127] Quemener A.M., Bachelot L., Forestier A., Donnou-Fournet E., Gilot D., Galibert M.D. (2020). The powerful world of antisense oligonucleotides: From bench to bedside. Wiley Interdiscip. Rev. RNA.

[128] Kim Y. (2023). Drug Discovery Perspectives of Antisense Oligonucleotides. Biomol. Ther..

[129] Huang S., Hao X.Y., Li Y.J., Wu J.Y., Xiang D.X., Luo S. (2022). Nonviral delivery systems for antisense oligonucleotide therapeutics. Biomater. Res..

[130] Ramasamy T., Ruttala H.B., Munusamy S., Chakraborty N., Kim J.O. (2022). Nano drug delivery systems for antisense oligonucleotides (ASO) therapeutics. J. Control. Release.

[131] Ruchi R., Raman G.M., Kumar V., Bahal R. (2025). Evolution of antisense oligonucleotides: Navigating nucleic acid chemistry and delivery challenges. Expert. Opin. Drug Discov..

[132] Kotula J.W., Pratico E.D., Ming X., Nakagawa O., Juliano R.L., Sullenger B.A. (2012). Aptamer-mediated delivery of splice-switching oligonucleotides to the nuclei of cancer cells. Nucleic Acid Ther..

[133] Wu C., Han D., Chen T., Peng L., Zhu G., You M., Qiu L., Sefah K., Zhang X., Tan W. (2013). Building a multifunctional aptamer-based DNA nanoassembly for targeted cancer therapy. J. Am. Chem. Soc..

[134] Li J., Zheng C., Cansiz S., Wu C., Xu J., Cui C., Liu Y., Hou W., Wang Y., Zhang L. (2015). Self-assembly of DNA nanohydrogels with controllable size and stimuli-responsive property for targeted gene regulation therapy. J. Am. Chem. Soc..

[135] Hong S., Sun N., Liu M., Wang J., Pei R. (2016). Building a chimera of aptamer–antisense oligonucleotide for silencing galectin-1 gene. RSC Adv..

[136] Dai Z., Li J., Lin Y., Wang Z., Huang Y. (2021). Facile Construction of a Solely-DNA-Based System for Targeted Delivery of Nucleic Acids. Nanomaterials.

[137] Luo F., Yang G., Bai X., Yuan D., Li L., Wang D., Lu X., Cheng Y., Wang Y., Song X. (2023). Anti-tumor effect of PD-L1-targeting antagonistic aptamer-ASO delivery system with dual inhibitory function in immunotherapy. Cell Chem. Biol..

[138] Yang G., Zhang S., Song W., Bai X., Li L., Luo F., Cheng Y., Wang D., Wang Y., Chen J. (2023). Efficient Targeted Delivery of Bifunctional Circular Aptamer-ASO Chimera to Suppress the SARS-CoV-2 Proliferation and Inflammation. Small.

[139] Thongchot S., Aksonnam K., Thuwajit P., Yenchitsomanus P.T., Thuwajit C. (2023). Nucleolin-based targeting strategies in cancer treatment: Focus on cancer immunotherapy (Review). Int. J. Mol. Med..

[140] Van den Avont A., Sharma-Walia N. (2023). Anti-nucleolin aptamer AS1411: An advancing therapeutic. Front. Mol. Biosci..

[141] Zhou Y.J., Li G., Wang J., Liu M., Wang Z., Song Y., Zhang X., Wang X. (2023). PD-L1: Expression regulation. Blood Sci..

[142] Wu Y., Chen W., Xu Z.P., Gu W. (2019). PD-L1 Distribution and Perspective for Cancer Immunotherapy-Blockade, Knockdown, or Inhibition. Front. Immunol..

[143] Komatsu S., Kitai H., Suzuki H.I. (2023). Network Regulation of microRNA Biogenesis and Target Interaction. Cells.

[144] Pagoni M., Cava C., Sideris D.C., Avgeris M., Zoumpourlis V., Michalopoulos I., Drakoulis N. (2023). miRNA-Based Technologies in Cancer Therapy. J. Pers. Med..

[145] Samad A.F.A., Kamaroddin M.F. (2023). Innovative approaches in transforming microRNAs into therapeutic tools. Wiley Interdiscip. Rev. RNA.

[146] Catuogno S., Di Martino M.T., Nuzzo S., Esposito C.L., Tassone P., de Franciscis V. (2019). An Anti-BCMA RNA Aptamer for miRNA Intracellular Delivery. Mol. Ther. Nucleic Acids.

[147] Catuogno S., Rienzo A., Di Vito A., Esposito C.L., de Franciscis V. (2015). Selective delivery of therapeutic single strand antimiRs by aptamer-based conjugates. J. Control. Release.

[148] Abnous K., Danesh N.M., Ramezani M., Alibolandi M., Bahreyni A., Lavaee P., Moosavian S.A., Taghdisi S.M. (2020). A smart ATP-responsive chemotherapy drug-free delivery system using a DNA nanostructure for synergistic treatment of breast cancer in vitro and in vivo. J. Drug Target..

[149] Ramezanpour M., Daei P., Tabarzad M., Khanaki K., Elmi A., Barati M. (2019). Preliminary study on the effect of nucleolin specific aptamer-miRNA let-7d chimera on Janus kinase-2 expression level and activity in gastric cancer (MKN-45) cells. Mol. Biol. Rep..

[150] Russo V., Paciocco A., Affinito A., Roscigno G., Fiore D., Palma F., Galasso M., Volinia S., Fiorelli A., Esposito C.L. (2018). Aptamer-miR-34c Conjugate Affects Cell Proliferation of Non-Small-Cell Lung Cancer Cells. Mol. Ther. Nucleic Acids.

[151] Esposito C.L., Nuzzo S., Kumar S.A., Rienzo A., Lawrence C.L., Pallini R., Shaw L., Alder J.E., Ricci-Vitiani L., Catuogno S. (2016). A combined microRNA-based targeted therapeutic approach to eradicate glioblastoma stem-like cells. J. Control. Release.

[152] Iaboni M., Russo V., Fontanella R., Roscigno G., Fiore D., Donnarumma E., Esposito C.L., Quintavalle C., Giangrande P.H., de Franciscis V. (2016). Aptamer-miRNA-212 Conjugate Sensitizes NSCLC Cells to TRAIL. Mol. Ther. Nucleic Acids.

[153] Esposito C.L., Cerchia L., Catuogno S., De Vita G., Dassie J.P., Santamaria G., Swiderski P., Condorelli G., Giangrande P.H., de Franciscis V. (2014). Multifunctional aptamer-miRNA conjugates for targeted cancer therapy. Mol. Ther..

[154] Zhang T., Wu Y., Yang D., Wu C., Li H. (2021). Preparation, characterization, and in vitro tumor-suppressive effect of anti-miR-21-equipped RNA nanoparticles. Biochem. Biophys. Res. Commun..

[155] Dai F., Zhang Y., Zhu X., Shan N., Chen Y. (2013). The anti-chemoresistant effect and mechanism of MUC1 aptamer-miR-29b chimera in ovarian cancer. Gynecol. Oncol..

[156] Nuzzo S., Catuogno S., Capuozzo M., Fiorelli A., Swiderski P., Boccella S., de Nigris F., Esposito C.L. (2019). Axl-Targeted Delivery of the Oncosuppressor miR-137 in Non-small-Cell Lung Cancer. Mol. Ther. Nucleic Acids.

[157] Li J., Qiu L., Xie S., Zhang J., Zhang L., Liu H., Li J., Zhang X., Tan W. (2018). Engineering a customized nanodrug delivery system at the cellular level for targeted cancer therapy. Sci. China Chem..

[158] Wu X., Ding B., Gao J., Wang H., Fan W., Wang X., Zhang W., Wang X., Ye L., Zhang M. (2011). Second-generation aptamer-conjugated PSMA-targeted delivery system for prostate cancer therapy. Int. J. Nanomed..

[159] Tanno T., Zhang P., Bailey C., Wang Y., Ittiprasert W., Devenport M., Zheng P., Liu Y. (2023). A novel aptamer-based small RNA delivery platform and its application to cancer therapy. Genes Dis..

[160] Haj-Yahia S., Nandi A., Benhamou R.I. (2023). Targeted Degradation of Structured RNAs via Ribonuclease-Targeting Chimeras (RiboTacs). Expert. Opin. Drug Discov..

[161] Dey S.K., Jaffrey S.R. (2019). RIBOTACs: Small Molecules Target RNA for Degradation. Cell Chem. Biol..

[162] Fang Y., Wu Q., Wang F., Liu Y., Zhang H., Yang C., Zhu Z. (2024). Aptamer-RIBOTAC Strategy Enabling Tumor-Specific Targeted Degradation of MicroRNA for Precise Cancer Therapy. Small Methods.

[163] Kwok A., Raulf N., Habib N. (2019). Developing small activating RNA as a therapeutic: Current challenges and promises. Ther. Deliv..

[164] Pandey S., Bednarz P.T., Oberli M.A., Veiseh O. (2024). Small activating RNA delivery in vivo: Challenges, prospects, and lessons learned from siRNA delivery. Nano Res..

[165] Tan C.P., Sinigaglia L., Gomez V., Nicholls J., Habib N.A. (2021). RNA Activation—A Novel Approach to Therapeutically Upregulate Gene Transcription. Molecules.

[166] Voutila J., Reebye V., Roberts T.C., Protopapa P., Andrikakou P., Blakey D.C., Habib R., Huber H., Saetrom P., Rossi J.J. (2017). Development and Mechanism of Small Activating RNA Targeting CEBPA, a Novel Therapeutic in Clinical Trials for Liver Cancer. Mol. Ther..

[167] Yoon S., Huang K.W., Reebye V., Mintz P., Tien Y.W., Lai H.S., Saetrom P., Reccia I., Swiderski P., Armstrong B. (2016). Targeted Delivery of C/EBPalpha -saRNA by Pancreatic Ductal Adenocarcinoma-specific RNA Aptamers Inhibits Tumor Growth In Vivo. Mol. Ther..

[168] Yoon S., Huang K.W., Andrikakou P., Vasconcelos D., Swiderski P., Reebye V., Sodergren M., Habib N., Rossi J.J. (2019). Targeted Delivery of C/EBPalpha-saRNA by RNA Aptamers Shows Anti-tumor Effects in a Mouse Model of Advanced PDAC. Mol. Ther. Nucleic Acids.

[169] Li C., Jiang W., Hu Q., Li L.C., Dong L., Chen R., Zhang Y., Tang Y., Thrasher J.B., Liu C.B. (2016). Enhancing DPYSL3 gene expression via a promoter-targeted small activating RNA approach suppresses cancer cell motility and metastasis. Oncotarget.

[170] Van Simaeys D., De La Fuente A., Zilio S., Zoso A., Kuznetsova V., Alcazar O., Buchwald P., Grilli A., Caroli J., Bicciato S. (2022). RNA aptamers specific for transmembrane p24 trafficking protein 6 and Clusterin for the targeted delivery of imaging reagents and RNA therapeutics to human beta cells. Nat. Commun..

[171] Porciani D., Tedeschi L., Marchetti L., Citti L., Piazza V., Beltram F., Signore G. (2015). Aptamer-Mediated Codelivery of Doxorubicin and NF-kappaB Decoy Enhances Chemosensitivity of Pancreatic Tumor Cells. Mol. Ther. Nucleic Acids.

[172] Hu J., Al-Waili D., Hassan A., Fan G.C., Xin M., Hao J. (2016). Inhibition of cerebral vascular inflammation by brain endothelium-targeted oligodeoxynucleotide complex. Neuroscience.

[173] Bu X., Wang L. (2025). Iron metabolism and the tumor microenvironment: A new perspective on cancer intervention and therapy (Review). Int. J. Mol. Med..

[174] Yoon S., Rossi J.J. (2017). Treatment of Pancreatic Cancer by Aptamer Conjugated C/EBPalpha-saRNA. Adv. Exp. Med. Biol..

[175] Mahjoubin-Tehran M., Atkin S.L., Bezsonov E.E., Jamialahmadi T., Sahebkar A. (2021). Harnessing the Therapeutic Potential of Decoys in Non-Atherosclerotic Cardiovascular Diseases: State of the Art. J. Cardiovasc. Dev. Dis..

[176] Yamakawa K., Nakano-Narusawa Y., Hashimoto N., Yokohira M., Matsuda Y. (2019). Development and Clinical Trials of Nucleic Acid Medicines for Pancreatic Cancer Treatment. Int. J. Mol. Sci..

[177] Datsyuk J.K., Paudel K.R., Rajput R., Kokkinis S., El Sherkawi T., Singh S.K., Gupta G., Chellappan D.K., Yeung S., Hansbro P.M. (2023). Emerging applications and prospects of NFkappaB decoy oligodeoxynucleotides in managing respiratory diseases. Chem.-Biol. Interact..

[178] Mehta M., Paudel K.R., Shukla S.D., Allam V., Kannaujiya V.K., Panth N., Das A., Parihar V.K., Chakraborty A., Ali M.K. (2021). Recent trends of NFkappaB decoy oligodeoxynucleotide-based nanotherapeutics in lung diseases. J. Control. Release.

[179] Yao X., Lyu P., Yoo K., Yadav M.K., Singh R., Atala A., Lu B. (2021). Engineered extracellular vesicles as versatile ribonucleoprotein delivery vehicles for efficient and safe CRISPR genome editing. J. Extracell. Vesicles.

[180] Wu H., Zhang L., Zhu Z., Ding C., Chen S., Liu R., Fan H., Chen Y., Li H. (2021). Novel CD123 polyaptamer hydrogel edited by Cas9/sgRNA for AML-targeted therapy. Drug Deliv..

[181] Han Y., Ding B., Zhao Z., Zhang H., Sun B., Zhao Y., Jiang L., Zhou J., Ding Y. (2018). Immune lipoprotein nanostructures inspired relay drug delivery for amplifying antitumor efficiency. Biomaterials.

[182] Wang D., Liu J., Duan J., Yi H., Liu J., Song H., Zhang Z., Shi J., Zhang K. (2023). Enrichment and sensing tumor cells by embedded immunomodulatory DNA hydrogel to inhibit postoperative tumor recurrence. Nat. Commun..

[183] Ma Y., Deng L., Li S. (2022). [Application of nanoparticles in CRISPR/Cas9-based gene therapy]. Sheng Wu Gong Cheng Xue Bao.

[184] Aljabali A.A.A., El-Tanani M., Tambuwala M.M. (2024). Principles of CRISPR-Cas9 technology: Advancements in genome editing and emerging trends in drug delivery. J. Drug Deliv. Sci. Technol..

[185] Razavi Z., Soltani M., Souri M., van Wijnen A.J. (2024). CRISPR innovations in tissue engineering and gene editing. Life Sci..

[186] Yang W., Yan J., Zhuang P., Ding T., Chen Y., Zhang Y., Zhang H., Cui W. (2022). Progress of delivery methods for CRISPR-Cas9. Expert. Opin. Drug Deliv..

[187] Whitley J.A., Cai H. (2023). Engineering extracellular vesicles to deliver CRISPR ribonucleoprotein for gene editing. J. Extracell. Vesicles.

[188] Lu Z., Yao X., Lyu P., Yadav M., Yoo K., Atala A., Lu B. (2021). Lentiviral Capsid-Mediated Streptococcus pyogenes Cas9 Ribonucleoprotein Delivery for Efficient and Safe Multiplex Genome Editing. CRISPR J..

[189] Dey S., Basu S., Ranjan A. (2023). Revisiting the Role of CD63 as Pro-Tumorigenic or Anti-Tumorigenic Tetraspanin in Cancers and its Theragnostic Implications. Adv. Biol..

[190] Lee J., Le Q.V., Yang G., Oh Y.K. (2019). Cas9-edited immune checkpoint blockade PD-1 DNA polyaptamer hydrogel for cancer immunotherapy. Biomaterials.

[191] Zhao J., Wang M., Yang Y., Wang G., Che F., Li Q., Zhang L. (2021). CD123 thioaptamer protects against sepsis via the blockade between IL-3/CD123 in a cecal ligation and puncture rat model. Nucleosides Nucleotides Nucleic Acids.

[192] Wang M., Wu H., Duan M., Yang Y., Wang G., Che F., Liu B., He W., Li Q., Zhang L. (2019). SS30, a novel thioaptamer targeting CD123, inhibits the growth of acute myeloid leukemia cells. Life Sci..

[193] Su-Tobon Q., Fan J., Goldstein M., Feeney K., Ren H., Autissier P., Wang P., Huang Y., Mohanty U., Niu J. (2025). CRISPR-Hybrid: A CRISPR-Mediated Intracellular Directed Evolution Platform for RNA Aptamers. Nat. Commun..

[194] Chen M., Huang X., Shi Y., Wang W., Huang Z., Tong Y., Zou X., Xu Y., Dai Z. (2024). CRISPR/Pepper-tDeg: A Live Imaging System Enables Non-Repetitive Genomic Locus Analysis with One Single-Guide RNA. Adv. Sci..

[195] Wang S., Su J.H., Zhang F., Zhuang X. (2016). An RNA-aptamer-based two-color CRISPR labeling system. Sci. Rep..

[196] Liu Y.J., Dou X.Q., Wang F., Zhang J., Wang X.L., Xu G.L., Xiang S.S., Gao X., Fu J., Song H.F. (2017). IL-4Ralpha aptamer-liposome-CpG oligodeoxynucleotides suppress tumour growth by targeting the tumour microenvironment. J. Drug Target..

[197] Lu D., Di Z., Li L., Zhao J., Zheng L. (2024). An aptamer-driven DNA nanodevice for improved delivery of synthetic immunostimulants. Nano Res..

[198] Sun L., Shen F., Tian L., Tao H., Xiong Z., Xu J., Liu Z. (2021). ATP-Responsive Smart Hydrogel Releasing Immune Adjuvant Synchronized with Repeated Chemotherapy or Radiotherapy to Boost Antitumor Immunity. Adv. Mater..

[199] Jiang T., Yang Z., Su Q., Fang L., Xiang Q., Tian C., Gao Q., Mao C., Huang C.Z., Zuo H. (2025). Bivalent OX40 Aptamer and CpG as Dual Agonists for Cancer Immunotherapy. ACS Appl. Mater. Interfaces.

[200] Wei H., Zhao Z., Wang Y., Zou J., Lin Q., Duan Y. (2019). One-Step Self-Assembly of Multifunctional DNA Nanohydrogels: An Enhanced and Harmless Strategy for Guiding Combined Antitumor Therapy. ACS Appl. Mater. Interfaces.

[201] Fan Q., Li Z., Yin J., Xie M., Cui M., Fan C., Wang L., Chao J. (2023). Inhalable pH-responsive DNA tetrahedron nanoplatform for boosting anti-tumor immune responses against metastatic lung cancer. Biomaterials.

[202] Wang D., Liu J., Duan J., Ma Y., Gao H., Zhang Z., Liu J., Shi J., Zhang K. (2022). Photocontrolled Spatiotemporal Delivery of DNA Immunomodulators for Enhancing Membrane-Targeted Tumor Photodynamic Immunotherapy. ACS Appl. Mater. Interfaces.

[203] Subramanian N., Kanwar J.R., Akilandeswari B., Kanwar R.K., Khetan V., Krishnakumar S. (2015). Chimeric nucleolin aptamer with survivin DNAzyme for cancer cell targeted delivery. Chem. Commun..

[204] Li X., Yang F., Zhou W., Yuan R., Xiang Y. (2020). Targeted and direct intracellular delivery of native DNAzymes enables highly specific gene silencing. Chem. Sci..

[205] Zhang K., Li Y., Liu J., Yang X., Xu Y., Shi J., Liu W., Li J. (2020). Y-Shaped Circular Aptamer–DNAzyme Conjugates for Highly Efficient in Vivo Gene Silencing. CCS Chem..

[206] Zheng J., Zhao S., Yu X., Huang S., Liu H.Y. (2017). Simultaneous targeting of CD44 and EpCAM with a bispecific aptamer effectively inhibits intraperitoneal ovarian cancer growth. Theranostics.

[207] Li X., Yang Y., Zhao H., Zhu T., Yang Z., Xu H., Fu Y., Lin F., Pan X., Li L. (2020). Enhanced in Vivo Blood-Brain Barrier Penetration by Circular Tau-Transferrin Receptor Bifunctional Aptamer for Tauopathy Therapy. J. Am. Chem. Soc..

[208] Zhang Z., Kuo J.C., Yao S., Zhang C., Khan H., Lee R.J. (2021). CpG Oligodeoxynucleotides for Anticancer Monotherapy from Preclinical Stages to Clinical Trials. Pharmaceutics.

[209] Kayraklioglu N., Horuluoglu B., Klinman D.M. (2021). CpG Oligonucleotides as Vaccine Adjuvants. Methods Mol. Biol..

[210] Zhang Z., Lu Y., Liu W., Huang Y. (2024). Nanomaterial-assisted delivery of CpG oligodeoxynucleotides for boosting cancer immunotherapy. J. Control. Release.

[211] Li M., Yao H., Yi K., Lao Y.H., Shao D., Tao Y. (2024). Emerging nanoparticle platforms for CpG oligonucleotide delivery. Biomater. Sci..

[212] Wang D., Huang J., Gui T., Yang Y., Feng T., Tzvetkov N.T., Xu T., Gai Z., Zhou Y., Zhang J. (2022). SR-BI as a target of natural products and its significance in cancer. Semin. Cancer Biol..

[213] Guan B., Zhang X. (2020). Aptamers as Versatile Ligands for Biomedical and Pharmaceutical Applications. Int. J. Nanomed..

[214] Xie S., Sun W., Fu T., Liu X., Chen P., Qiu L., Qu F., Tan W. (2023). Aptamer-Based Targeted Delivery of Functional Nucleic Acids. J. Am. Chem. Soc..

[215] Dhanya C.R., Mary A.S., Madhavan M. (2023). Aptamer-siRNA chimeras: Promising tools for targeting HER2 signaling in cancer. Chem. Biol. Drug Des..

[216] Thomas B.J., Porciani D., Burke D.H. (2022). Cancer immunomodulation using bispecific aptamers. Mol. Ther. Nucleic Acids.

[217] Peng X., Liu Y., Peng F., Wang T., Cheng Z., Chen Q., Li M., Xu L., Man Y., Zhang Z. (2024). Aptamer-controlled stimuli-responsive drug release. Int. J. Biol. Macromol..

[218] Chung Y.D., Tsai Y.C., Wang C.H., Lee G.B. (2025). Aptamer selection via versatile microfluidic platforms and their diverse applications. Lab Chip.

[219] Komarova N., Barkova D., Kuznetsov A. (2020). Implementation of High-Throughput Sequencing (HTS) in Aptamer Selection Technology. Int. J. Mol. Sci..

[220] Zhu C., Feng Z., Qin H., Chen L., Yan M., Li L., Qu F. (2024). Recent progress of SELEX methods for screening nucleic acid aptamers. Talanta.

[221] Kruspe S., Giangrande P.H. (2017). Aptamer-siRNA Chimeras: Discovery, Progress, and Future Prospects. Biomedicines.

